# Subspecialization of Surgical Specialties in the US

**DOI:** 10.1001/jamahealthforum.2025.3192

**Published:** 2025-09-19

**Authors:** René Karadakic, David C. Chan, Bruce E. Landon, Nancy L. Keating, Christopher Manz, Jukka-Pekka Onnela, Thomas C. Tsai, Yuhua Zhang, Michael L. Barnett

**Affiliations:** 1 Department of Health Policy and Management, Harvard T.H. Chan School of Public Health, Boston, Massachusetts; 2 Department of Medical Oncology, Dana-Farber Cancer Institute, Boston, Massachusetts; 3 Haas School of Business, Berkley, California; 4 Department of Veterans Affairs, Palo Alto, California; 5 Department of Health Care Policy, Harvard Medical School, Boston, Massachusetts; 6 Division of General Internal Medicine, Department of Medicine, Beth Israel Deaconess Medical Center, Boston, Massachusetts; 7 Division of General Internal Medicine, Department of Medicine, Brigham and Women’s Hospital, Boston, Massachusetts; 8 Department of Surgery, Brigham and Women’s Hospital, Boston, Massachusetts; 9 Department of Biostatistics, Harvard T.H. Chan School of Public Health, Boston, Massachusetts

## Abstract

**Importance:**

Subspecialists—physicians with narrower clinical focus—play an increasing role in US health care, particularly in surgery, where most trainees now pursue fellowship training. Yet little is known about the degree of subspecialization among practicing surgeons and the evolving role of surgical generalists.

**Objective:**

To quantify trends in surgical subspecialization and examine the geographic distribution of subspecialists and the procedural scope of surgical generalists.

**Design, Setting, and Participants:**

A retrospective cohort study using 100% Medicare Part B data from 2000, 2010, and 2021, including all fee-for-service beneficiaries treated by physicians in general surgery, neurosurgery, ophthalmology, orthopedic surgery, and otolaryngology was caried out. A novel classification method combining k-means clustering, large language models, and expert validation to distinguish subspecialists from surgical generalists based on procedural claims was used. The analysis was undertaken in 2023 and 2024.

**Main Outcomes and Measures:**

The number of distinct subspecialties, the share of subspecialists in each surgical specialty, their geographic distribution across hospital referral regions (HRRs), and the association between subspecialist supply and procedural diversity among generalists.

**Results:**

Overall, more than 70 000 surgeons were included. The share of subspecialists increased from 38% in 2000 to 58% in 2021, reflecting the growth of recognized subspecialties (from 24 to 33) and expansion of existing ones. Subspecialization rates varied by specialty, rising from 9% to 28% in otolaryngology and from 66% to 77% in neurosurgery. While subspecialist supply per 100 000 fee-for-service beneficiaries remained stable, the mean number of surgical generalists per 100 000 declined from 105 to 50. A 10% increase in subspecialist supply in an HRR was associated with a 0.94% decrease (95% CI, –0.15% to –0.41%) in the number of unique procedures performed by generalist surgeons.

**Conclusions and Relevance:**

This cohort study found that subspecialization has considerably reshaped the surgical workforce, concentrating care among subspecialists while narrowing the procedural scope of generalist surgeons. These shifts raise concerns about access to generalist care, particularly in regions with declining generalist supply, and suggest the need for policies that consider both specialization and geographic equity in surgical workforce planning.

## Key Points

**Question:**

How has surgical subspecialization evolved in the US, and what are its implications for generalist surgeons and geographic access to care?

**Findings:**

In this retrospective cohort study of more than 70 000 surgeons using Medicare data from 2000 to 2021, the share of subspecialists rose from 38% to 58%. Subspecialist growth was associated with reduced procedural scope among generalists and substantial geographic variation in access.

**Meaning:**

Surgical subspecialization has expanded considerably, narrowing generalist roles and contributing to uneven access to specialized care across regions.

## Teaser

This cohort study examines trends in surgical subspecialization in 5 surgical fields, the geographic distribution of subspecialists, and the procedural scope of surgical generalists.

## Introduction

Over the past 4 decades, there has been a dramatic increase in specialization among physicians in the US.^1^ In 1980, 62% of office visits for older adults were to primary care physicians; by 2013, most office visits were to specialists.^2^ Over the same period, the American Board of Medical Specialties markedly increased its accredited specialties, with the number of subspecialty board certifications expanding from 28 in 1980 to 147 by 2020.^3^ This transformation reflects the rise of subspecialization in the US, whereby physicians increasingly focus on narrower clinical areas as a response to the growing complexity of care—particularly for an aging population—and rapidly advancing medical technology.^3^ Concurrently, a rising number of resident physicians are opting to subspecialize.^4,5^ Subspecialization could play a vital role in advancing care for specific conditions, but their growing prominence raises concerns about fragmented care, overdiagnosis, and differences in access to specialized care.^6,7,8,9,10,11^

Subspecialization has particularly important implications for the surgical workforce,^12^ for which there is the most clearly established relationship between specific procedural volume and outcomes of care,^13,14^ and where training is highly dependent on an apprenticeship model of clinical fellowship training following residency.^15,16^ Moreover, in recent decades multiple general surgery subspecialties have branched into independent training pathways, such as vascular, thoracic, and plastic surgery.^17,18^ This rise in surgical subspecialization is redefining the prominence and role of surgical generalists who do not pursue additional subspecialization. Given the length and intensity of surgical training, the degree of subspecialization and geographic distribution of surgical subspecialists may significantly influence differences in access to surgical care across the US.

Despite the rapid growth of subspecialization, comprehensive evidence on its workforce trends and broader implications remains limited.^19^ To address this gap, this study applied machine learning and expert validation to Medicare claims data for 2000, 2010, and 2021, enabling the classification of subspecialties within 5 major surgical specialties over 2 decades. By examining the geographic distribution of surgical generalists and subspecialists and assessing the effects of subspecialization on surgical generalists’ practice scope, this study provides critical insights into the evolving dynamics of surgeon specialization in the US.

## Methods

### Study Population and Data Source

The Harvard T.H. Chan School of Public Health institutional review board approved the study with a waiver of informed consent. Reporting followed the Strengthening the Reporting of Observational Studies in Epidemiology (STROBE) reporting guidelines. This retrospective cohort study analyzed 100% Medicare Part B fee-for-service data from 2000, 2010, and 2021 to examine trends in subspecialization across 5 major surgical specialties. For 2021, we used the provider-by-services public use file published by the Centers for Medicare & Medicaid Services (CMS).^20^ For 2000 and 2010, we constructed equivalent files accessed through the Virtual Research Data Center (VRDC), ensuring consistency by replicating the 2021 public use file. In addition, we obtained physicians’ graduation year and medical school from public directories.^21^ Overall, the final sample included 19 665 general surgeons, 3274 neurosurgeons, 16 781 ophthalmologists, 22 025 orthopedic surgeons, and 8117 otolaryngologists in 2021 (eTable 2 in Supplement 1).

We used CMS specialty codes^22^ to define physicians in general surgery, neurosurgery, ophthalmology, orthopedic surgery, and otolaryngology, based on their initial surgical specialty residency training (eTable 1 in Supplement 1). Together, these specialties encompassed 70 015 physicians in 2021, representing 12.3% of the US specialist physician workforce.^23^ Within each of these broad specialties, we then additionally included surgeons who underwent further subspecialty training that only can be pursued after completing these residencies, as opposed to the complement of surgeons who remained surgical generalists, who pursued no further training after their residencies. For example, colorectal surgery and thoracic surgery were grouped under “general surgery” because these are clinical fellowships that follow general surgery residency.^24^ Similarly, hand surgeons were grouped under orthopedic surgery. We distinguished between surgical generalists, who trained beginning with a general surgery residency, from surgical generalists within each of the specialties we studied who trained in that specialty without either pursuing further subspecialty training or limiting their practice to a specific clinical area.

The study population included all fee-for-service Medicare beneficiaries who received any services from surgeons in the 5 broad specialties or relevant subspecialties in 2000, 2010, and 2021. Services were identified in the Carrier file using Healthcare Common Procedure Coding System (HCPCS) codes; we also documented spending for each HCPCS code based on the average Medicare allowed amount across all claims. To comply with privacy regulations, physician-HCPCS code cells with fewer than 11 beneficiaries were excluded. To optimize clustering and focus on procedures, we excluded surgeons who billed exclusively for evaluation and management (E&M) codes. Although these surgeons comprised 15% of all surgeons, they accounted for less than 1% of all services and revenue.

### Clustering Analysis and Labeling

**Figure 1.**
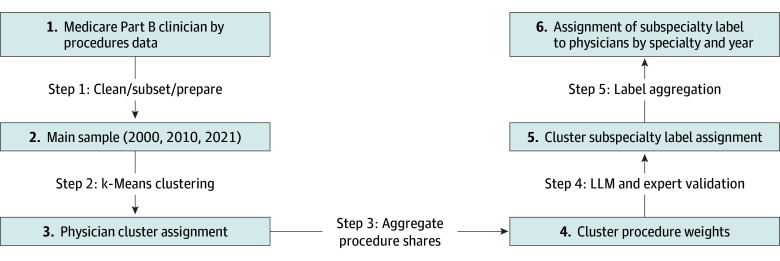
Flowchart of the Subspecialty Detection Method The subspecialty detection pipeline and relevant steps taken to classify physicians into distinct subspecialties are shown. Detailed descriptions of each step and additional information are provided in Supplement 1. LLM indicates large language model.

We developed novel methods for detecting subspecialization beyond formal subspecialty training, using k-means clustering enhanced by large language models (LLMs) and expert validation.^25,26^ For each physician and year, we calculated revenue shares for all non-E&M procedure codes to capture the proportion of revenue from each procedure relative to a physician’s total billing (Figure 1, step 1). K-means clustering grouped physicians based on the similarity of their procedure revenue shares (Figure 1, step 2), with the number of clusters aligned to observed subspecialties in the National Health Care Provider Taxonomy.^27^

The importance of procedures within each cluster, derived from procedure shares (Figure 1, step 3), was used to generate preliminary subspecialty labels through computational support from ChatGPT-4 (OpenAI),^26^ followed by expert validation (M.L.B., B.E.L., N.L.K.; Figure 1, step 4). Final labels were refined by experts, and synonymous clusters were aggregated (eg, “spine surgery,” “surgery of the spine”). Physicians not fitting a specific subspecialty were labeled as surgical generalists within their specialty (Figure 1, step 5). Additional details of the subspecialty detection method are provided in the eMethods in Supplement 1. The analysis was undertaken in 2023 and 2024.

### Outcomes

We quantified multiple outcomes to capture subspecialization patterns, including the number of distinct subspecialties, the proportion of subspecialists within each specialty, and their geographic distribution across hospital referral regions (HRRs). We also examined the association between subspecialist growth and generalist growth within specialties, as well as their relationship with overall fee-for-service population growth across HRRs from 2000 to 2021.

To assess procedural diversity among surgical generalists, we measured the number of unique procedures performed and the concentration of procedure revenue using the Herfindahl-Hirschman Index (HHI). The HHI is calculated as the sum of squared revenue shares across procedures; a value of 1 indicates complete concentration in 1 procedure, whereas values closer to 0 reflect a broader and more evenly distributed procedure mix.

### Covariates

We included key physician characteristics to contextualize subspecialization trends: year of graduation from medical school, physician specialty label, subspecialty labels, and Hospital Referral Region (HRR) based on physician zip codes; each physician was assigned to their modal zip code in Medicare Part B claims in each year.

### Statistical Analysis

Descriptive statistics summarized baseline physician characteristics and subspecialization trends. Multiple validation tests ensured the robustness of subspecialty detection, including external benchmarking, physician switching analysis, and t-distributed stochastic neighbor embedding (t-SNE)^28^ for visual validation (Supplement 1).

t-SNE was used to create 2-dimensional maps of physicians based on procedure shares, allowing visual comparison of cluster-based subspecialty labels with external classifications, such as Medicare-determined subspecialty labels (eTable 1 in Supplement 1). A 2021 t-SNE visualization (eFigure 1 in Supplement 1) showed clear separation between specialties, with overlaps primarily among physicians without distinct procedural specialization or from specialties with shared procedural domains (eg, orthopedic spine surgery and spinal neurosurgery).

The association between subspecialization and surgical generalists’ practice scope (unique procedures or procedure HHI) was assessed using log-log regression models,^29^ with subspecialists per 100 000 fee-for-service enrollees as the key independent variable. Models controlled for generalist supply, fee-for-service population size, and included specialty, year, and state fixed effects (full details in Supplement 1). Analyses were conducted in R statistical software (version 4.2.2; R Foundation).

## Results

### Subspecialty Detection and Trends in Subspecialization

The number of physicians in the main sample increased from 60 054 in 2000 to 69 835 in 2021, with notable variation across specialties. Using cluster-based subspecialty labels, the number of unique subspecialties increased from 24 in 2000 to 33 in 2021, with otolaryngology and neurosurgery each adding 4 new subspecialties (eTable 3 in Supplement 1). In contrast, Medicare-determined subspecialty labels identified only 11 subspecialties in 2000 and 12 in 2021.

Cluster-based subspecialty labels reclassified many physicians from generalists to subspecialists over time. In 2000, 38% of physicians were identified as subspecialists by cluster-based subspecialty labels compared with only 10% by Medicare-determined subspecialty labels; by 2021, cluster-based subspecialty labels identified 58% as subspecialists, whereas Medicare-determined subspecialty labels identified only 16% (eTable 4 in Supplement 1). Cluster-based subspecialty labels provided greater granularity, accurately capturing clinical distinctions and correcting misclassifications found in Medicare-determined subspecialty labels (eFigures 2 and 3 in Supplement 1) and NPPES labels (eFigures 4 to 8 in Supplement 1).

**Figure 2.**
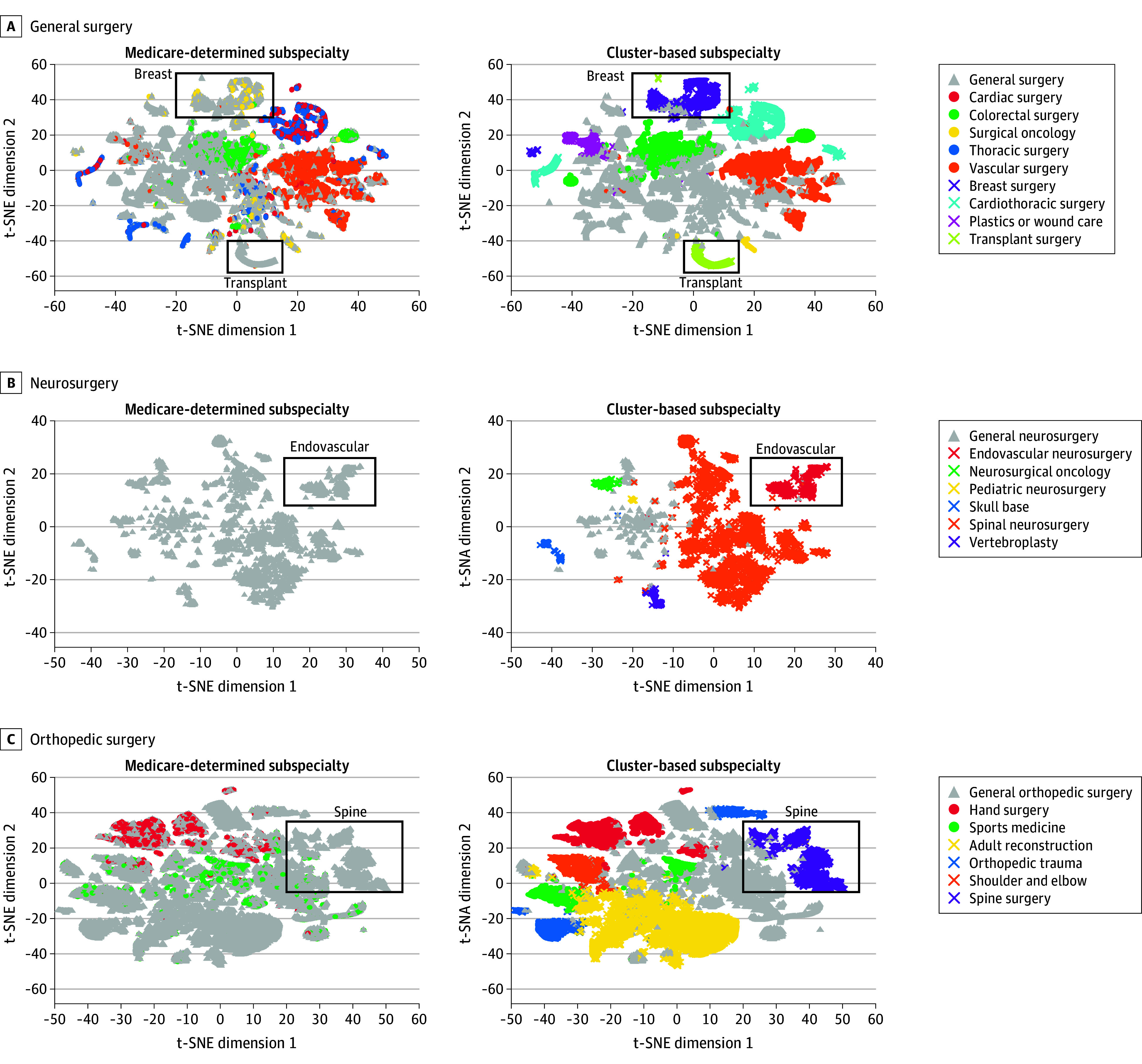
t-Distributed Stochastic Neighbor Embedding (t-SNE) Representation of Physicians by Subspecialty Labels in 2021 (A) The t-SNE visualizations of physicians’ procedure shares in general surgery, (B) neurosurgery, and (C) orthopedic surgery. Different colors represent the assigned subspecialty labels, obtained either from our cluster-based subspecialty labels (CBS) on the right or from the Medicare-determined subspecialty labels (MDS) on the left. The general category of a subspecialty (eg, general surgery) is indicated with light gray triangles. Subspecialties that are identified using only MDS or MDS and cluster-based subspecialty labels have a dot as indicator. Subspecialties only identified using cluster-based subspecialty labels have a cross as indicator. For improved visibility we added annotations to distinct clusters, which are mentioned in the Results section of the article or simply to enhance readability of the figure.

Cluster-based subspecialty labels also offered more precise differentiation within individual specialties, as visualized using t-SNE (Figure 2). For example, breast surgery formed a distinct cluster under cluster-based subspecialty labels, whereas Medicare-determined subspecialty labels blended these physicians with general surgeons and surgical oncologists (Figure 2A). Similar improvements were seen for transplant surgery within general surgery and spine surgery among orthopedic surgeons, where cluster-based subspecialty labels delineated sharper subspecialty boundaries (Figure 2, B and C). One example of greater clarity was hand surgery, where cluster-based subspecialty labels showed that 48.8% of all hand surgeons were labeled as general orthopedists by Medicare labels (1138 of 2329; eFigure 3 in Supplement 1). Neurosurgery exhibited the clearest distinction between subspecialties, highlighting cluster-based subspecialty labels’ robustness (Figure 2B). Additional t-SNE visualizations and methodologic details are provided in eFigures 9 to 13 in Supplement 1.

**Figure 3.**
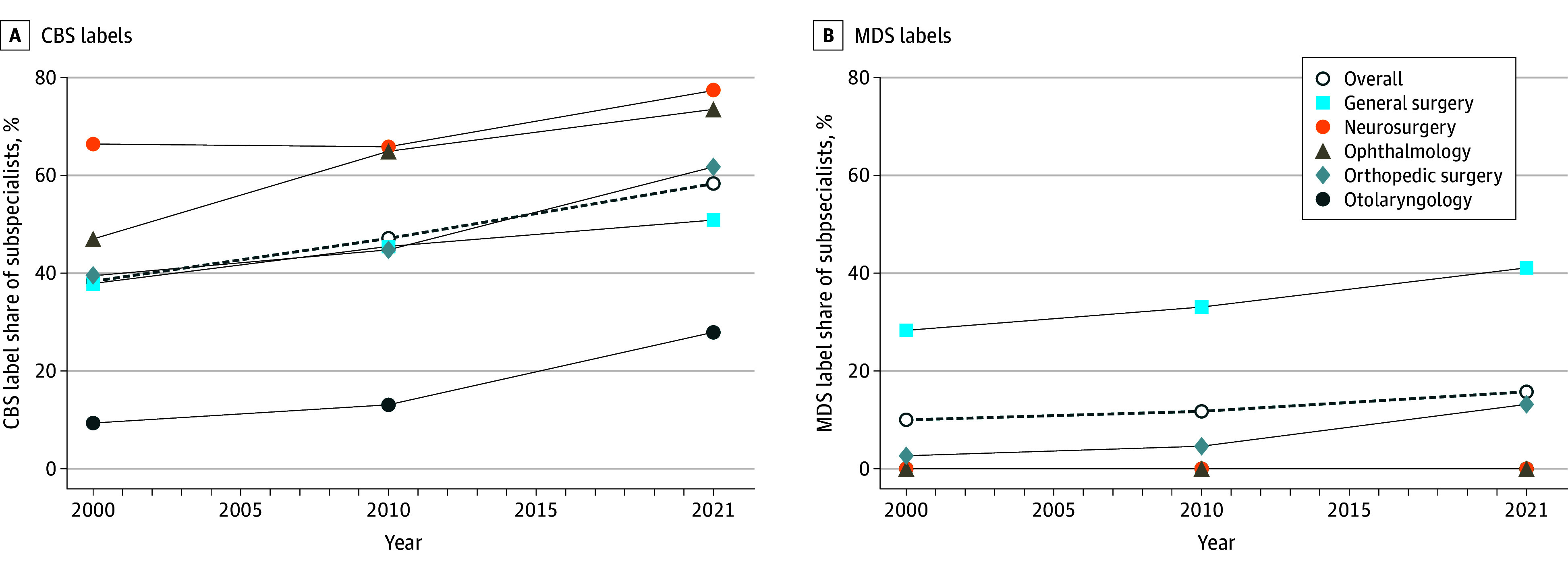
Trends in Subspecialization by Specialty and Year, Cluster-Based (CBS) vs Medicare-Determined (MDS) Subspecialty Labels The share of physicians classified as subspecialists separately by year and specialty are presented. Each dot represents the share of all subspecialists among all physicians within the specialty. Each line indicates the subspecialization share for a different specialty indicated by different colors and markers, the overall group consists of the subspecialization share for the entire sample across all specialties. Note that neurosurgery, ophthalmology and otolaryngology do not have any subspecialty labels in Medicare data.

Subspecialization increased across all 5 specialties using cluster-based subspecialty labels, with the largest relative growth in otolaryngology, where the share of subspecialists rose from 9% in 2000 to 28% in 2021 (Figure 3). Neurosurgery, already highly subspecialized, saw modest growth (66% to 77%). In general surgery, the rise in subspecialists was driven by a slight increase in subspecialists and a large decline in generalist surgeons between 2010 and 2021 (eTable 5 in Supplement 1). Notably there was a substantial difference in both baseline levels of subspecialization as well as trends in subspecialization overall and within specialties when comparing subspecialty shares using cluster-based compared with Medicare-determined subspecialty labels.

### Subspecialization and Physician Education and Training

More recent medical school cohorts exhibited a higher share of subspecialists than graduates of less recent cohorts. For the 1975 to 1979 cohorts, only 53% of graduates still practicing in 2021 were classified as subspecialists, vs 65% of graduates in the 2005 to 2010 cohorts (eFigure 3 in Supplement 1). This trend was particularly pronounced in neurosurgery (63% to 84% for 1975-1979 and 2005-2010, respectively), ophthalmology (62% in 1975-1979 to 86% in 2005-2010), and orthopedic surgery (56% in 1975-1979 to 68% in 2005-2010), while general surgery and otolaryngology showed more stable trends, with smaller increases in the share of subspecialists for the most recent graduation cohorts.

### Geographic Variation in Subspecialization

**Figure 4.**
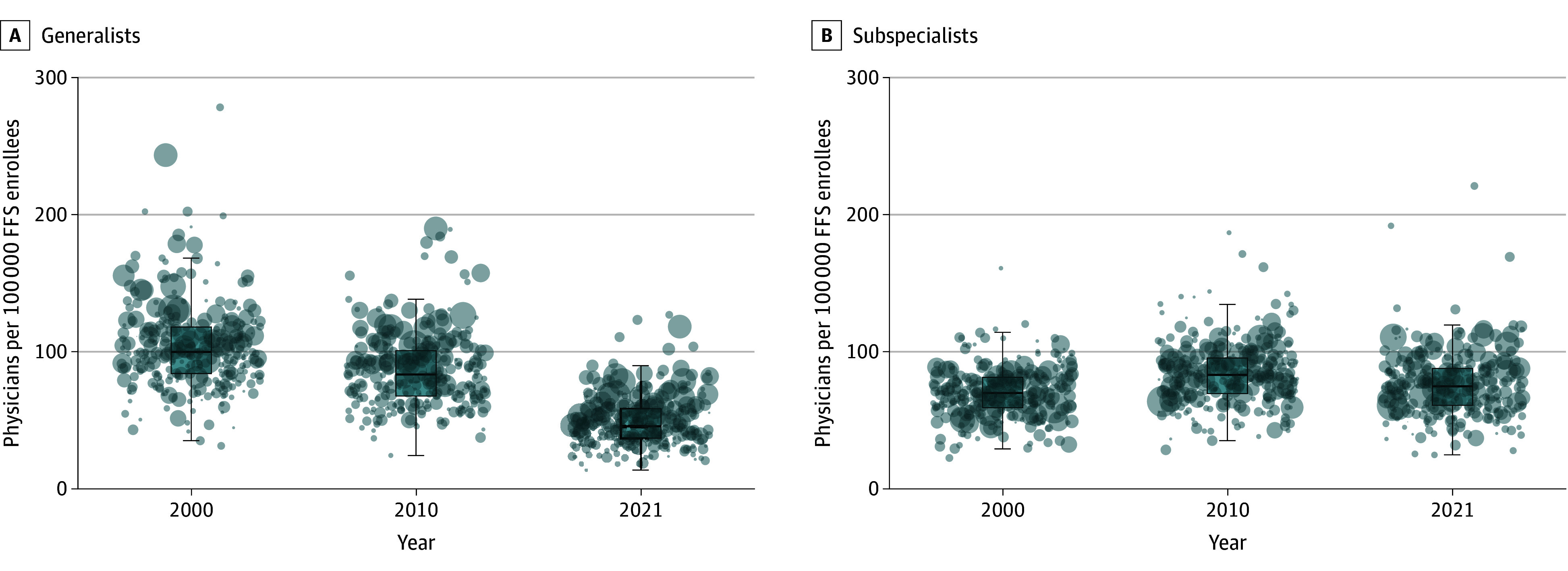
Geographic Variation of Physicians Across Hospital Referral Regions (HRRs) Over Time Boxplots and jitter plots of generalist and subspecialist physicians per 100 000 fee-for-service Medicare enrollees across HRRs for 2000, 2010, and 2021. All 5 surgical specialty groups (general surgery, neurosurgery, ophthalmology, orthopedic surgery, otolaryngology) are included. Each bubble represents an HRR. The bubble size is proportional to the size of HRRs in 2000. Subspecialist and surgical generalists were classified using the cluster-based subspecialty labels. The boxes indicate interquartile range (IQR); lines within boxes indicate medians; whiskers extend to 1.5 × IQR.

Using cluster-based subspecialty labels, in 2000, there was considerable variation in the number of surgical generalists and subspecialists per 100 000 Medicare fee-for-service enrollees (Figure 4). By 2021, the number of surgical generalists per 100 000 beneficiaries declined from 105 to 50, whereas subspecialist supply remained stable, a pattern repeated across each of the individual subspecialties (eFigure 4 in Supplement 1). The decline in surgical generalists was driven by a 56% increase in the Medicare fee-for-service population (eFigure 5 in Supplement 1) and a 21% reduction in the number of surgical generalists across the 5 specialties. This decline was also observed when using Medicare-determined subspecialty labels, with far lower numbers of subspecialists (eFigure 6 in Supplement 1). Physician supply was strongly positively associated with HRR size (eFigure 7 in Supplement 1). Subspecialist numbers per 100 000 fee-for-service enrollees grew in parallel with population growth, maintaining a stable supply across HRRs over time.

In 2021, substantial variation persisted in subspecialist supply across the 50 largest HRRs, ranging from 38 to 117 subspecialists per 100 000 beneficiaries, reflecting differences in access even among similarly sized regions (eFigure 8 in Supplement 1). Baseline surgeon supply in 2000 was not associated with subsequent changes in the subspecialist share, except in neurosurgery and otolaryngology, where higher initial supply weakly correlated with greater growth in the share of subspecialists (eFigures 9-10 in Supplement 1).

### Scope of Practice of Generalist Surgeons

In adjusted regression models, there was a statistically significant association between changes in the number of subspecialist per 100 000 fee-for-service Medicare beneficiaries and the scope of practice for surgical generalists (eTable 6 in Supplement 1). Specifically, a 10% increase in subspecialist supply per 100 000 beneficiaries was associated with a decrease of 1.18 unique procedure codes billed by generalist surgeons (95% CI: −0.15 to −0.04), a 0.94% relative reduction (Table; eTable 7 in Supplement 1). Similarly, there was an increase in the concentration of procedure revenue among surgeons, as measured by the HHI. A 10% increase in the number of subspecialists per 100 000 fee-for-service enrollees was associated with an average 0.53% increase in the HHI (95% CI, 0.01 to 0.09). Compared with the average HHI of 0.23 among all surgeons studied, this corresponds to an increase in HHI of 0.01, analogous to the difference in HHI between the 60th and 50th percentiles in the overall HHI distribution.

**Table.** Association Between Subspecialist Supply and Measures of Surgical Generalists Scope of Practice^a^

Variable	No. of unique procedures per surgeon		HHI (concentration of procedure revenue)	
	Excluding fixed effects	Including fixed effects	Excluding fixed effects	Including fixed effects
Baseline mean value	12.65	12.65	0.23	0.23
Effect of 10% increase in subspecialists per 100 000 (95% CI)	−1.88 (−1.65 to −1.33)	−1.19 (−1.48 to −0.41)	0.04 (1.44 to 1.70)	0.01 (0.13 to 0.92)
Relative change per 10% increase, %	−1.49	−0.94	1.57	0.53
No. of observations	102 013	102 013	101 772	101 772
*R* ^2^	0.003	0.11	0.006	0.10
Standard errors	HC1	By state	HC1	By state
Fixed effects: state	NA	Yes	NA	Yes
Fixed effects: year	NA	Yes	NA	Yes
Fixed effects: specialty	NA	Yes	NA	Yes

Abbreviations: HC1, heteroskedasticity robust standard errors with degrees of freedom adjustment; HHI; Herfindahl-Hirschman Index; NA, not applicable.

^a^ The Table presents the results of ordinary least squares regressions of our outcomes on the number of subspecialists per 100 000 fee-for-service enrollees (columns 1 and 3). The simple model columns (1-3) present results of simple ordinary least squares regressions without additional controls and fixed effects. All 5 surgical specialty groups (general surgery, neurosurgery, ophthalmology, orthopedic surgery, otolaryngology) are included. Effect sizes are expressed as effects relative to the mean of the respective outcome variables. Results for our main specification are presented in columns 2 and 4 and include various controls as well as fixed effects for specialty, year, and state. 95% CIs are constructed using heteroskedasticity robust standard errors and where applicable we cluster standard errors at the state level. Raw regression output can be found in eTable 7 in Supplement 1.

## Discussion

The findings of this cohort study suggest that subspecialization within surgical specialties, identified through empirically-derived labels, has substantially reshaped the US surgical workforce over the past 2 decades. By 2021, subspecialists comprised most surgeons in all specialties except otolaryngology, with more recent graduates increasingly likely to subspecialize.^30^ To our knowledge, this is the first national analysis of subspecialization trends across multiple surgical specialties. We also found that existing administrative subspecialty labels fail to accurately capture physician subspecialization compared with our novel approach, which integrates Medicare claims data, machine learning, and expert validation. Advances in technology, changing patient demographics, and a growing surgical knowledge base likely contribute to the rise of surgical subspecialization, alongside a notable decline in surgical generalists. Geographic variation in subspecialization was striking, with up to a 4-fold difference across the 50 largest health care markets. These findings highlight the growing prominence of subspecialists while raising questions about their impact on access to care and the evolving role of generalist surgeons.

We also found that substantial variation in subspecialist supply across similarly sized HRRs may change generalist practice.^30^ Higher subspecialist density was linked to fewer unique procedures and greater procedural volume concentration among surgical generalists. As subspecialists dominate complex or high-volume procedures, generalists may face a narrowing scope of practice. However, this could also drive generalists to specialize within a smaller set of procedures, promoting focused expertise, particularly in specialty-dense regions. It is not yet clear whether this shift in surgical generalist practice has positive or negative implications for patient care or the physician workforce. Our findings align with earlier concerns about the declining general surgery workforce, including prior work showing that reduced generalist supply may affect access to emergency and broad-scope surgical care, particularly in rural or resource-constrained settings.^31^ This shift highlights the need for workforce planning efforts that consider the complementary roles of generalists and subspecialists in meeting diverse population health needs. Although our study did not examine other fields, it is plausible that subspecialization is more advanced in surgery than in less procedure-oriented specialties, where subspecialist roles may be defined by diagnostic focus, prescribing patterns, or care settings rather than by procedural volume.

Our findings also highlight the limitations of current specialty classification systems in health care data. Inconsistent use of available taxonomies can lead to inaccurate classifications in public physician directories,^32^ impacting both policy decisions as well as data available for individual decision making. In congressional policy, the 2019 Resident Physician Shortage Reduction Act proposed graduate medical education (GME) funding allocations based on outdated specialty classifications identifying only 18 specialties (vs 142 board certified specialties and subspecialties in 2008).^33,34^ Network adequacy policy in Medicare Advantage^35^ and Medicaid^36^ also accounts for a small fraction of the existing diversity of subspecialties. More precise data on clinical expertise is needed to guide policy and improve access to surgical care.

Although this study did not assess the impact of subspecialist supply on care quality, the differences in distribution raise concerns about equitable access to specialized surgical care. Given the well-established volume-outcomes relationship in surgery, future research should explore whether HRRs with fewer subspecialists—and potentially lower volumes for specialized procedures—have different outcomes than those with higher subspecialist density.^37,38,39,40,41^ Such differences may contribute to documented gaps in surgical care quality between rural and urban areas. Even in larger health care markets, variation in the surgical workforce could influence outcomes. As more residents subspecialize and surgical generalists retire, these gaps may widen, restricting access to advanced care and worsening geographic inequities.

### Limitations

This cohort study had several limitations. First, it relies on fee-for-service Medicare data, which may limit generalizability to privately insured or Medicare Advantage populations. It also excludes specialties with limited relevance to the Medicare population, such as cosmetic and plastic surgery and subspecialties that blur the distinction between generalist and subspecialist roles (eg, emergency general surgery). Second, the focus on procedural aspects of practice does not account for surgeon’s differences in diseases treated or prescribing, potentially providing an incomplete view of their broader roles. Third, despite improvements over existing classification methods, the absence of a gold standard for physician classification limits definitive categorization. Fourth, this study did not assess the potential drivers of specialization, such as changes in the patient population, the fee schedule, or other systemic incentives. Finally, the study focused on surgical specialties. Future research adapting these approaches to other fields, such as oncology or cardiology, will gain additional insights into workforce evolution and drivers of specialization.

## Conclusions

Subspecialization has profoundly transformed the US surgical workforce in recent decades, enhancing surgical expertise but potentially narrowing the procedural scope of generalist surgeons and expanding geographic differences in access to specialized care. In some specialties, such as neurosurgery and otolaryngology, greater subspecialization was associated with higher baseline surgeon supply. Limitations in existing subspecialist classification systems raise concerns about the ability of policymakers to accurately identify workforce gaps. Although subspecialists play a vital role in advancing surgical care, their concentration in larger health care markets raises important questions about the value of highly specialized training and its necessity for optimal public health. Although the impact of subspecialization on patient outcomes remains uncertain, integrating subspecialization considerations into health policy is essential to improve workforce distribution and enhance care delivery.

## Notes

Supplement 1. eMethods eFigure 1. t-SNE Representation of Physicians by Specialty 2021 eFigure 2. Medicare Fee-For-Service Population Growth eFigure 3. Share of Subspecialists by Graduation Year in 2021 eFigure 4. Geographic Variation of Physicians across HRRs, Specialties and Time eFigure 5. Geographic Variation of Physicians across HRRs over Time with Constant Fee-For-Service Population eFigure 6. Geographic Variation of Physicians across HRRs over Time with Constant Fee-For-Service Population (Medicare-Determined Subspecialty Labels) eFigure 7. Association between Surgical Generalists/Subspecialists and Fee-for-Service Population within HRRs from 2000, 2010 and 2021 eFigure 8. Geographic Variation of Subspecialists across 50 Largest HRRs eFigure 9. Change in Subspecialist Share from 2000 to 2021 relative to the Number of Surgeons per 100,000 FFS Enrollees across HRRs eFigure 10. Change in Subspecialist Share relative to the Number of Surgeons per 100,000 FFS Enrollees, by Specialty across HRRs eTable 1. Specialty Codes Defining Inclusion of Physicians in Main Sample eTable 2. Summary Statistics of Main Sample of Physicians by Specialty and Year eTable 3. Trends in the Number of Distinct Subspecialties eTable 4. Share of Physicians Reclassified by Specialty and Year eTable 5. Subspecialists and Generalists by Label Type and Year eTable 6. Summary Statistics of Main Estimation Sample eTable 7. Association of Subspecialists per FFS Beneficiary with Measures of Generalists Scope of Practice (Raw Regression Output) eReferences

Supplement 2. Data Sharing Statement
