# Errors in Supplement 1

**DOI:** 10.1001/jamahealthforum.2026.1696

**Published:** 2026-05-15

In the Original Investigation titled “Subspecialization of Surgical Specialties in the US,”^1^ published online September 19, 2025, there were errors in Supplement 1. The eFigures were updated. This article was corrected online.
