# Supplementary material for: Subspecialization of Surgical Specialties in the US
Source: JAMA Health Forum. 2025 Sep 19;6(9):e253192. doi: 10.1001/jamahealthforum.2025.3192 (PMC12449727; doi:10.1001/jamahealthforum.2025.3192)
Supplement: Supplement 1. — eMethods eFigure 1. t-SNE Representation of Physicians by Specialty 2021 eFigure 2. Medicare Fee-For-Service Population Growth eFigure 3. Share of Subspecialists by Graduation Year in 2021 eFigure 4. Geographic Variation of Physicians across HRRs, Specialties and Time eFigure 5. Geographic Variation of Physicians across HRRs over Time with Constant Fee-For-Service Population eFigure 6. Geographic Variation of Physicians across HRRs over Time with Constant Fee-For-Service Population (Medicare-Determined Subspecialty Labels) eFigure 7. Association between Surgical Generalists/Subspecialists and Fee-for-Service Population within HRRs from 2000, 2010 and 2021 eFigure 8. Geographic Variation of Subspecialists across 50 Largest HRRs eFigure 9. Change in Subspecialist Share from 2000 to 2021 relative to the Number of Surgeons per 100,000 FFS Enrollees across HRRs eFigure 10. Change in Subspecialist Share relative to the Number of Surgeons per 100,000 FFS Enrollees, by Specialty across HRRs eTable 1. Specialty Codes Defining Inclusion of Physicians in Main Sample eTable 2. Summary Statistics of Main Sample of Physicians by Specialty and Year eTable 3. Trends in the Number of Distinct Subspecialties eTable 4. Share of Physicians Reclassified by Specialty and Year eTable 5. Subspecialists and Generalists by Label Type and Year eTable 6. Summary Statistics of Main Estimation Sample eTable 7. Association of Subspecialists per FFS Beneficiary with Measures of Generalists Scope of Practice (Raw Regression Output) eReferences [file jamahealthforum-e253192-s001.pdf]

Supplemental Online Content

Karadakic R, Chan DC, Landon BE, et al. Subspecialization of surgical specialties in the US. *JAMA Health Forum*. 2025;6(9):e253192. doi:10.1001/jamahealthforum.2025.3192

eMethods.
eFigure 1. t-SNE Representation of Physicians by Specialty 2021
eFigure 2. Medicare Fee-For-Service Population Growth
eFigure 3. Share of Subspecialists by Graduation Year in 2021
eFigure 4. Geographic Variation of Physicians across HRRs, Specialties and Time
eFigure 5. Geographic Variation of Physicians across HRRs over Time with Constant Fee-For-Service Population
eFigure 6. Geographic Variation of Physicians across HRRs over Time with Constant Fee-For-Service Population (Medicare-Determined Subspecialty Labels)
eFigure 7. Association between Surgical Generalists/Subspecialists and Fee-for-Service Population within HRRs from 2000, 2010 and 2021
eFigure 8. Geographic Variation of Subspecialists across 50 Largest HRRs
eFigure 9. Change in Subspecialist Share from 2000 to 2021 relative to the Number of Surgeons per 100,000 FFS Enrollees across HRRs
eFigure 10. Change in Subspecialist Share relative to the Number of Surgeons per 100,000 FFS Enrollees, by Specialty across HRRs
eTable 1. Specialty Codes Defining Inclusion of Physicians in Main Sample
eTable 2. Summary Statistics of Main Sample of Physicians by Specialty and Year
eTable 3. Trends in the Number of Distinct Subspecialties
eTable 4. Share of Physicians Reclassified by Specialty and Year
eTable 5. Subspecialists and Generalists by Label Type and Year
eTable 6. Summary Statistics of Main Estimation Sample
eTable 7. Association of Subspecialists per FFS Beneficiary with Measures of Generalists Scope of Practice (Raw Regression Output)
eReferences.

This supplemental material has been provided by the authors to give readers additional information about their work.

© 2025 Karadakic R, et al. *JAMA Health Forum*.

eMethods.

Subspecialty Detection Method

This section provides a detailed description of the method used to classify physicians into subspecialties. The process is summarized in the flowchart presented in Figure M1. We describe each step of the method in detail below, outlining the procedures and criteria applied at each stage.

Figure M1: Flowchart of Subspecialty Detection Method

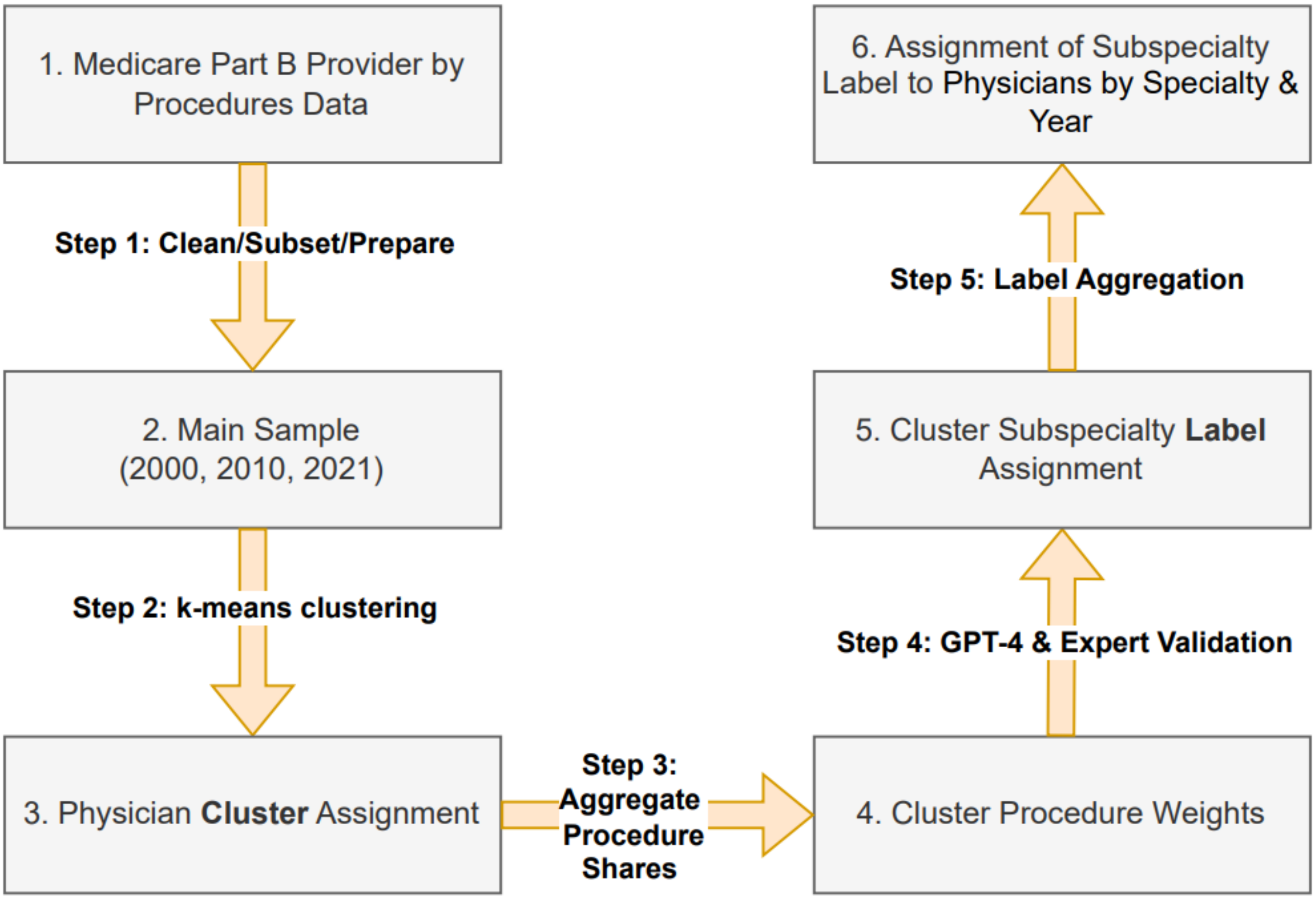

Notes: The figure shows the subspecialty detection pipeline and steps taken to classify physicians into distinct subspecialties.

Data Preparation (Step 1)

To identify subspecialties within the five major surgical specialties, we conducted a comprehensive analysis of Medicare Part B claims data, focusing on physician revenue shares from non–Evaluation and Management (E&M) procedures. Rather than emphasizing the overall volume of procedures, this approach concentrated on the procedural mix, capturing the relative distribution of specific types of procedures performed by physicians. This distinction allowed us to differentiate between surgical generalists and subspecialists based on the diversity of their medical practices.

In this framework, specialization is not determined by the absolute revenue a provider generates from a given procedure but by the proportional contribution of that procedure to their overall services. Total revenue is constructed by multiplying the total services of a provider for a procedure with the average allowed amount of the procedure. The share of procedure p for physician i expressed as γ_{ip} and is defined as the fraction of revenue τ of procedure p over the sum of all of physician i's procedure revenue:

$$\gamma_{ip} = \frac{\tau_{ip}}{\sum_p \tau_{ip}}$$

© 2025 Karadakic R, et al. *JAMA Health Forum*.

Clustering Approach (Step 2)

Once procedure shares were calculated, we used k-means clustering to cluster physicians into distinct groups. This algorithm partitions providers into k clusters by iteratively assigning each physician to the cluster with the closest mean as measured by the Euclidean distance of their procedure shares γ_{ip}. To ensure that clusters accurately captured procedural focus within a specific context, the algorithm was applied separately for each specialty and each year. This approach mitigated potential confounding effects from changes in procedure codes over time and enhanced computational efficiency.

Table M1: Hyperparameter Selection by Specialty

**Specialty**	**Number of Clusters (k)**
General Surgery	20
Neurosurgery	10
Ophthalmology	12
Orthopedic Surgery	16
Otolaryngology	16

Notes: The table provides the selected number of clusters which were used to initialize the k-means clustering algorithm for each specialty.

Since k-means is an unsupervised machine learning algorithm and does not rely on ground truth information about cluster assignments or physician subspecialties, determining the appropriate number of clusters required careful consideration. For each specialty, we experimented with various numbers of clusters, guided by the observed number of medical subspecialties listed in the National Plan and Provider Enumeration System (NPPES), which is determined by the National Uniform Claim Committee.[1] To enhance cluster differentiation, we initially set the number of clusters to double the observed number of subspecialties, as shown in Table M1. We ran the algorithm until convergence and allowed for a maximum of 300 iterations until convergence.

It is important to note that the clusters identified by the algorithm do not directly correspond to clinical subspecialties. Instead, the algorithm groups providers into distinct clusters based on their procedure shares. As a result, clusters may reflect differences in procedural focus that do not necessarily align with formal subspecialty distinctions. For example, in general surgery, the algorithm frequently identified two distinct groups: one performing a high proportion of gallbladder procedures and the other focusing on hernia repairs. While these groups were distinct, they both fall within the scope of general surgery rather than representing separate clinical subspecialties. The process of assigning clinical labels to these clusters is a separate step, which will be detailed further below.

Cluster Aggregation (Step 3)

Once k-means had assigned physicians to clusters, we aggregated the procedure shares, γ_{ip}, for all physicians within a cluster across all procedures and constructed procedure share weights which are defined as follows:

$$\lambda_{cp} = \frac{\sum_i \gamma_{icp}}{\sum_p \sum_i \gamma_{icp}}$$

Here γ_{icp} corresponds to the procedure shares from step one, subset to the relevant physicians in cluster c. Next, λ_{cp} is the procedure weight of procedure p within cluster c, which was constructed as the sum of all procedure shares over all physicians i in each cluster c, divided by the total sum of all procedure shares over all procedures and physicians in the cluster. This provides us with a weight of how proportionally important a given procedure is for a respective cluster. Table M2 provides an overview of what this looks like for a cluster that in the final step was labeled as breast surgery.

© 2025 Karadakic R, et al. *JAMA Health Forum.*

Table M2: Cluster Procedure Weights for Breast Surgery Cluster

HCPCS Code	Description	Share	Cumulative
99999	E & M	0.425	0.425
19301	Partial removal of breast	0.344	0.769
38525	Biopsy or removal of lymph nodes of neck	0.078	0.847
19303	Total removal of breast	0.042	0.899
38900	Lymph node imaging during surgery	0.038	0.927
19125	Removal of breast growth	0.010	0.937
19083	Biopsy of breast accessed through the skin with ultrasound guidance	0.007	0.944

Notes: The table provides the cluster procedure weights for a cluster which later was labeled as breast surgery.

Labeling Approach (Step 4)

Once k-means clustering assigned physicians to specific clusters and cluster procedure weights were calculated, the next step in our subspecialty identification process was assigning appropriate subspecialty labels to each cluster. This labeling process combined expert input from medical professionals (MLB, BEL, NLK) with support from advanced language models, specifically OpenAI's ChatGPT-4.

To assign subspecialty labels, we analyzed the procedure shares of physicians within each cluster and aggregated these to construct procedure weights for the cluster (see previous section). These weights reflect the relative proportion of each procedure billed by physicians in the cluster. Frequently billed procedures were assigned higher weights, while less frequently billed procedures received lower weights.

For example, Table M2 lists the top procedures and their respective weights for a cluster labeled as breast surgery. Procedures such as breast removals and lymph node interventions had the highest weights in this cluster (other than E&M codes). To generate initial labels for each cluster, we utilized ChatGPT-4 through the Linktransformer interface,[2] using the following prompt:

"*You are a medical expert on medical subspecialization with extensive knowledge of Medicare claims data. What subspecialty within general surgery would predominantly bill the following procedures, including the procedure shares in parentheses? Respond with the subspecialty label only, no additional text. If the provider is not subspecialized, respond with the general category.*"

We provided ChatGPT-4 with a list of cluster-specific procedure descriptions and their corresponding weights, ensuring that the procedural mix informed the output. This enabled the model to make fine-grained distinctions between clusters based on their procedural focus.

The cluster labels generated by ChatGPT-4 were reviewed and refined by experts with extensive experience in health services research and clinical practice. This iterative process ensured that the final labels accurately reflected the procedural characteristics of each cluster and aligned with established medical subspecialty classifications.

Cluster Aggregation (Step 5)

After all clusters were labeled, those with the same label were aggregated to create a cohesive categorization of subspecialties. For instance, in the clustering of general surgery physicians in 2021, we initially identified 20 clusters. Among these, five were labeled as general surgery, three as cardiothoracic surgery, three as colorectal surgery, one as plastic/wound care surgery, five as vascular surgery, one as breast surgery, one as surgical oncology, and one as transplant surgery. This aggregation process facilitated a more organized and clinically relevant categorization of subspecialties within each specialty, ensuring that distinctions across clusters did not obscure meaningful clinical distinctions.

© 2025 Karadakic R, et al. *JAMA Health Forum*.

For example, within the general surgery specialty, several clusters were distinct based on procedural differences, such as one cluster predominantly performing hernia procedures and another focused on gallbladder procedures. While these clusters are separated due to variations in procedural mix, they do not represent distinct clinical subspecialties and were therefore grouped under general surgery.

Additionally, clusters with slightly varying labels, such as “spinal surgery” and “surgery of the spine,” were combined into a single subspecialty category to ensure consistency and clarity in subspecialty labeling. This step allowed for a comprehensive and standardized representation of subspecialties across specialties and years.

Label Validation

We employed multiple methods to validate our cluster-based subspecialty (CBS) labels following the k-means clustering and expert labeling process. First, we benchmarked our cluster-based subspecialty labels against existing subspecialty labels available in Medicare data, derived from the CMS Healthcare Provider Taxonomy,[3] which we refer to as Medicare-determined subspecialty (MDS) labels. Second, we tracked physicians longitudinally to assess the stability of their subspecialty labels over time. Frequent transitions between distinct subspecialties could suggest issues with the clustering process, as highly subspecialized physicians are unlikely to switch subspecialties due to the significant costs associated with additional training and practice changes. Third, we compared our labels with those in the National Plan and Provider Enumeration System (NPPES). Although NPPES data is self-reported and incomplete, there should be reasonable alignment between our cluster-based subspecialty labels and the reported subspecializations where applicable. Finally, we used t-distributed stochastic neighbor embedding (t-SNE) to visually evaluate the clustering results relative to other labeling systems. This technique provided a visual representation of the clusters, helping to confirm the distinctiveness and coherence of the identified subspecialties.

Label Validation Medicare-Determined versus Cluster-Based Subspecialty Labels

Validation of cluster-based subspecialty labels against Medicare-determined subspecialty labels in Medicare data is limited to general surgery and orthopedic surgery, as Medicare-determined subspecialty labels are unavailable for neurosurgery, ophthalmology, and otolaryngology. Despite this constraint, comparing cluster-based subspecialty labels with existing Medicare-determined subspecialty labels for general surgery and orthopedic surgery provides a meaningful assessment of the reliability and accuracy of our labeling method.

© 2025 Karadakic R, et al. *JAMA Health Forum*.

Figure M2: Label Validation General Surgery - Medicare-Determined versus Cluster-Based Subspecialty Labels 2021

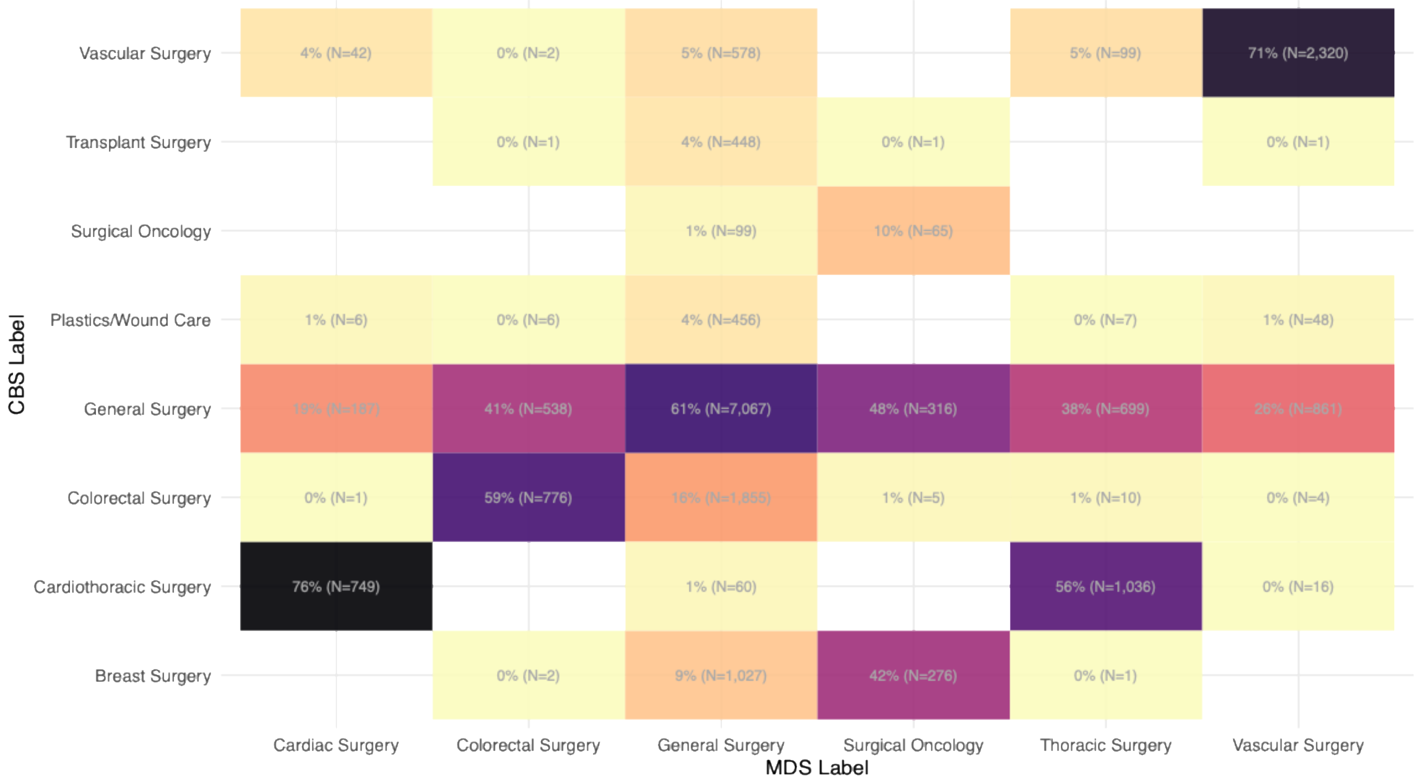

Notes: This figure shows the proportion of physicians with a specific cluster-based subspecialty label as a fraction of all physicians with a respective Medicare-determined subspecialty label. The x-axis depicts Medicare-determined subspecialty labels for general surgery in 2021, while the y-axis includes all distinct cluster-based subspecialty labels for the year 2021. For example, the top-left cell indicates that 4% (N=42) of physicians with the Medicare-determined subspecialty label "Cardiac Surgery" are labeled as "Vascular Surgery" using cluster-based subspecialty labels. Blank cells do not include any physicians.

Figure M2 depicts the proportion of physicians with a given Medicare-determined subspecialty label who fall within a specific cluster-based subspecialty label. For example, in the top-right cell, 71% (N=2,320) of general surgery physicians in 2021 with the Medicare-determined subspecialty label "Vascular Surgery" were also assigned the cluster-based subspecialty label "Vascular Surgery." In this figure, a darker color represents a higher proportion of physicians with a Medicare-determined subspecialty label aligning with the corresponding cluster-based subspecialty label, while a lighter color indicates a lower proportion.

Overall, Figure M2 demonstrates substantial overlap between Medicare-determined subspecialty and cluster-based subspecialty labels. Most physicians with a specific Medicare-determined subspecialty label were assigned a corresponding cluster-based subspecialty label (e.g., vascular surgery). However, perfect alignment was not observed. Some physicians with a general surgery Medicare-determined subspecialty label were assigned subspecialty cluster-based subspecialty labels, and conversely, certain physicians with subspecialty Medicare-determined subspecialty labels were categorized as surgical generalists in our data. These discrepancies suggest that the algorithm identified some physicians as likely mislabeled subspecialists and others as potentially mislabeled surgical generalists under the Medicare-determined subspecialty system.

Figure M3: Label Validation Orthopedic Surgery - Medicare-Determined versus Cluster-Based Subspecialty Labels 2021

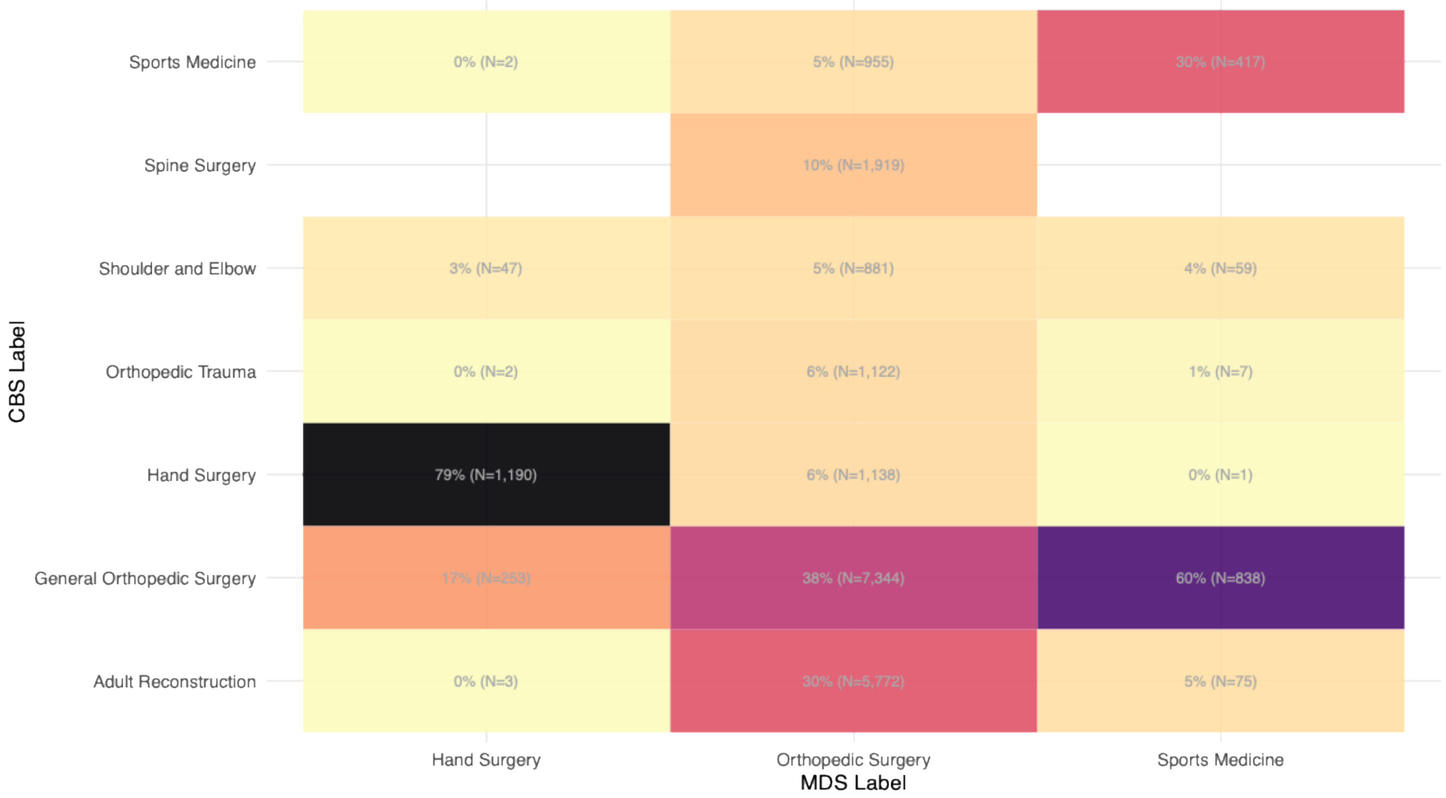

Notes: For orthopedic surgery, a similar pattern emerged. For example, 79% of individuals with a hand surgery Medicare-determined subspecialty label were classified as hand surgeons using our algorithm, with only a small fraction of 21% not falling into this subspecialty. However, a significant number of physicians labeled as general orthopedic surgeons (N=1,138) according to the Medicare determined subspecialty labels were classified as hand surgeons using our cluster-based subspecialty labels. This suggests a notable discrepancy between the reported subspecialty labels in the Medicare-determined subspecialty system and those identified through our cluster-based subspecialty labeling method. Blank cells do not include any physicians.

For orthopedic surgery, a similar pattern emerged. For example, 79% of individuals with a hand surgery MDS label were classified as hand surgeons using our algorithm, with only a small fraction of 21% not falling into this subspecialty. However, a significant number of physicians labeled as general orthopedic surgeons (N=1,138) according to the MDS labels were classified as hand surgeons using our CBS labels. This suggests a notable discrepancy between the reported subspecialty labels in the MDS system and those identified through our CBS labeling method.

Physician Tracking over Time

The second validation exercise tracks physicians over time to assess the extent to which they transition between different subspecialties. This analysis is limited to physicians observed in both 2010 and 2021. Although extending the analysis to earlier data, such as 2000, is possible, practical challenges arise. The transition from the Unique Provider Identification Number (UPIN) system to the National Provider Identifier (NPI) system in 2007 complicates the creation of a reliable crosswalk over time. Additionally, significant turnover in the physician workforce over a 20-year period would substantially reduce the sample of physicians consistently observed between 2000 and 2020, limiting the robustness of such an analysis. For these reasons, we report physician label transitions only from 2010 to 2021.

If our algorithm accurately identifies subspecialties, we would expect few physicians to transition between clinically distinct subspecialties (e.g., from hand surgery to spine surgery), as such shifts typically require significant changes in practice patterns, training, and education. The high opportunity costs associated with retraining make these transitions rare. However, switches between closely related subspecialties, which are not clinically distinct, may still occur. Additionally, given the trend toward increased subspecialization, some physicians may transition from surgical generalist roles within a specialty to more specialized roles, or

© 2025 Karadakic R, et al. *JAMA Health Forum*.

vice versa. Table M3 provides an overview of these transitions across all specialties, detailing the proportion of physicians and their subspecialty switching behavior.

Overall, more than 55% of physicians across all specialties maintained their subspecialization over the 11-year period. Between 11% (in ophthalmology) and 27% (in orthopedic surgery) transitioned from being surgical generalists within their specialty to subspecialists, while 6% to 14% moved in the opposite direction. A small fraction, up to 6% in neurosurgery and orthopedic surgery, switched between distinct subspecialties. Importantly, these transitions rarely involved shifts to clinically unrelated subspecialties, suggesting that significant switching between distinct subspecialties over time is uncommon.

Table M3: Cluster-Based Subspecialty Label Changes by Specialty, 2010 to 2021

	No Change	Change to Subspecialist	Change to Generalist	Change Subspecialty
General Surgery	68% (7,546)	16% (1,781)	14% (1,501)	3% (285)
Neurosurgery	66% (1,154)	17% (298)	11% (188)	6% (107)
Ophthalmology	71% (8,192)	11% (1,298)	14% (1,584)	3% (401)
Orthopedic Surgery	55% (7,155)	27% (3,480)	13% (1,666)	6% (790)
Otolaryngology	75% (3,952)	19% (996)	6% (299)	1% (41)

Notes: This table provides the share of physicians in each specialty and summarizes changes in their respective subspecialty labels. The sample consists of physicians present in both 2010 and 2021. Column 1 indicates the specialty. Column 2 shows the share of physicians in each specialty whose subspecialty label remained unchanged between 2010 and 2021. Column 3 reports the share of physicians who transitioned from being labeled as surgical generalists to a subspecialty. Column 4 reflects the share of physicians who held a subspecialty label in 2010 but were classified as surgical generalists in 2021. Column 5 represents the share of physicians whose classification changed from one distinct subspecialty to another (e.g., from vascular surgery to cardiothoracic surgery).

Label Comparison NPPES versus Cluster-Based Subspecialty Labels

To validate our subspecialty labels, we compared them with the classifications and specializations reported in the National Plan and Provider Enumeration System (NPPES), widely regarded as the most comprehensive registry of medical specializations. While a notable limitation of NPPES is that many physicians either fail to update their subspecialization information or lack reported specialization details altogether, this comparison still provides valuable insights and helps assess the quality of our subspecialty classification.

Below, we present comparison figures between the NPPES labels and cluster-based subspecialty labels. NPPES includes two separate sets of labels: classification and specialization. In our 2021 sample, more than 99% of physicians had a reported classification label, whereas only 17% had a reported specialization label. To ensure a meaningful comparison, we combined the specialization and classification labels, excluding and merging groups with very small physician counts. This approach allowed for a more comprehensive validation of our subspecialty identification process, ensuring that our method aligns with external standards while maintaining clarity in visual presentation.

The main conclusion from these figures is that our cluster-based subspecialty labels generally align well with detailed NPPES subspecialty labels when available. However, we also found evidence of significant lack of specificity within the NPPES data. This suggests potential inaccuracies in NPPES labels, reinforcing that while our cluster-based subspecialty labeling provides a reliable classification system, external NPPES data may contain inconsistencies that should be considered when interpreting the alignment between the two sets of labels.

© 2025 Karadakic R, et al. *JAMA Health Forum*.

Figure M4: General Surgery 2021 – NPPES versus Cluster-Based Subspecialty Labels

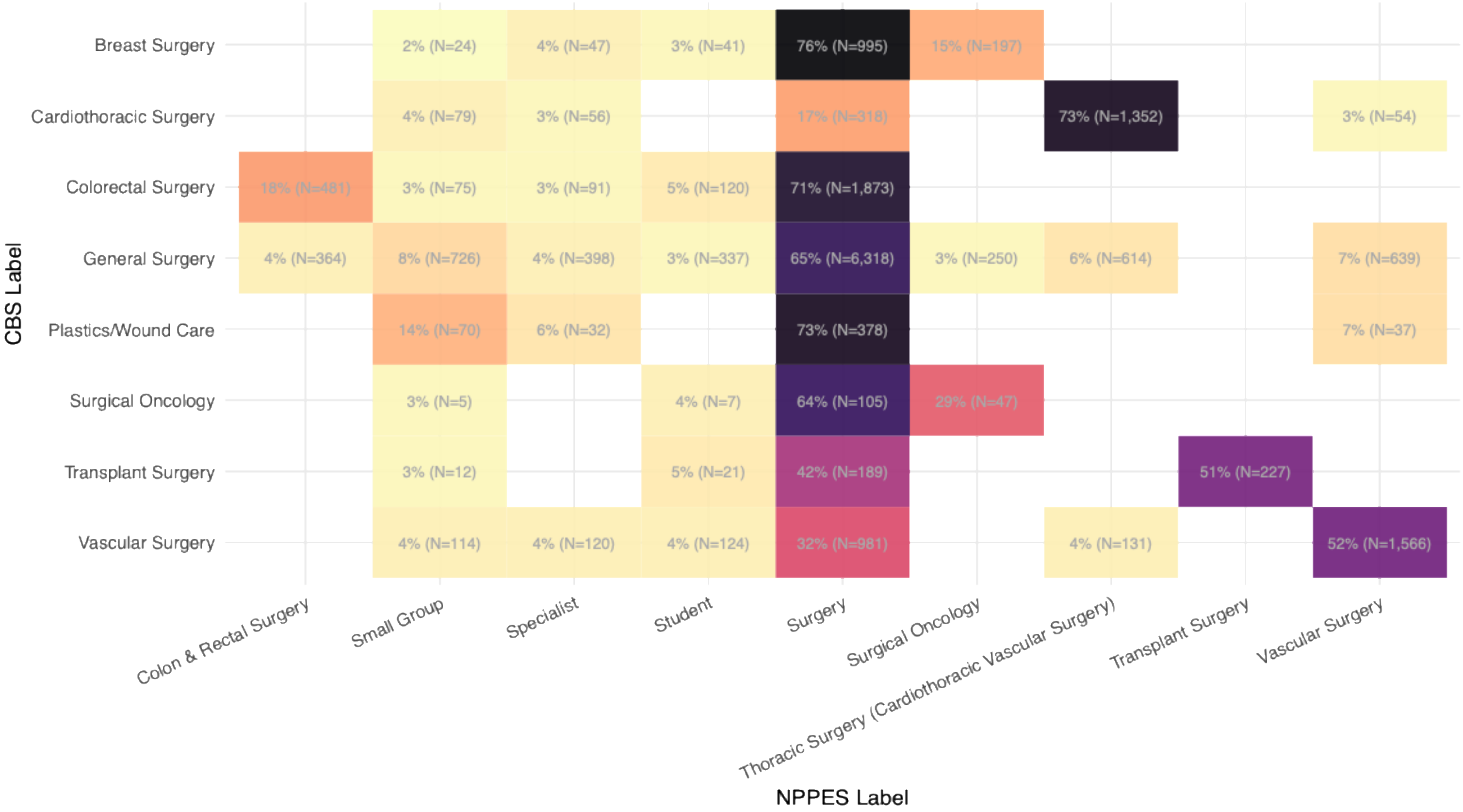

Notes: This figure shows the proportion of physicians with a specific cluster-based subspecialty label as a fraction of all physicians with a respective National Plan and Provider Enumeration System (NPPES) label. The x-axis depicts NPPES labels for general surgery in 2021, while the y-axis includes all distinct cluster-based subspecialty labels in 2021. For example, the bottom-right cell indicates that 52% (N=1,566) of physicians with the NPPES label "Vascular Surgery" were also labeled as "Vascular Surgery" using cluster-based subspecialty labels. Blank cells do not include any physicians. Blank cells do not include any physicians.

Figure M5: Neurosurgery 2021 – NPPES versus Cluster-Based Subspecialty Labels

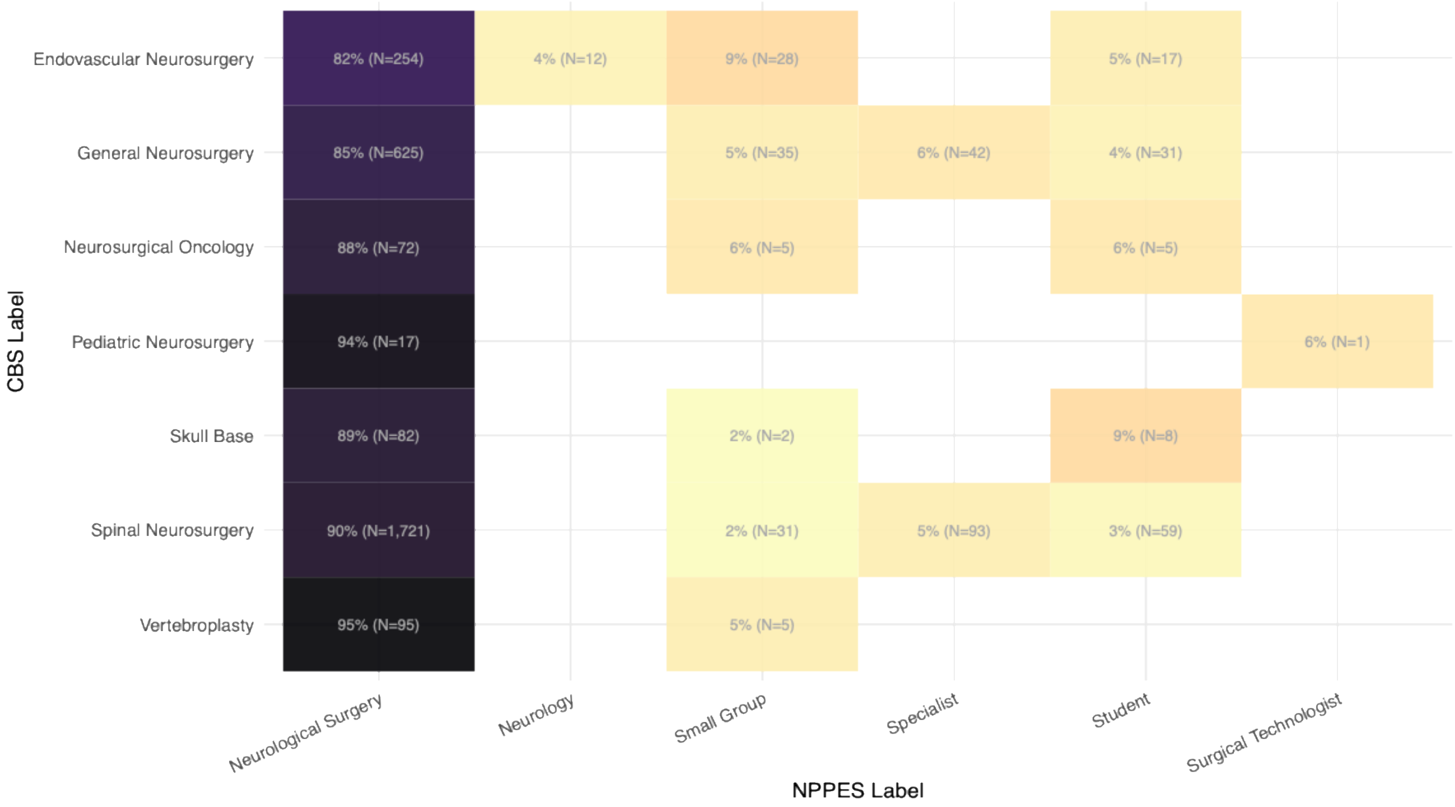

© 2025 Karadakic R, et al. *JAMA Health Forum*.

Notes: This figure shows the proportion of physicians with a specific cluster-based subspecialty label as a fraction of all physicians with a respective National Plan and Provider Enumeration System (NPPES) label. The x-axis depicts NPPES labels for neurosurgery in 2021, while the y-axis includes all distinct cluster-based subspecialty labels in 2021. Blank cells do not include any physicians.

Figure M6: Ophthalmology 2021 – NPPES versus Cluster-Based Subspecialty Labels

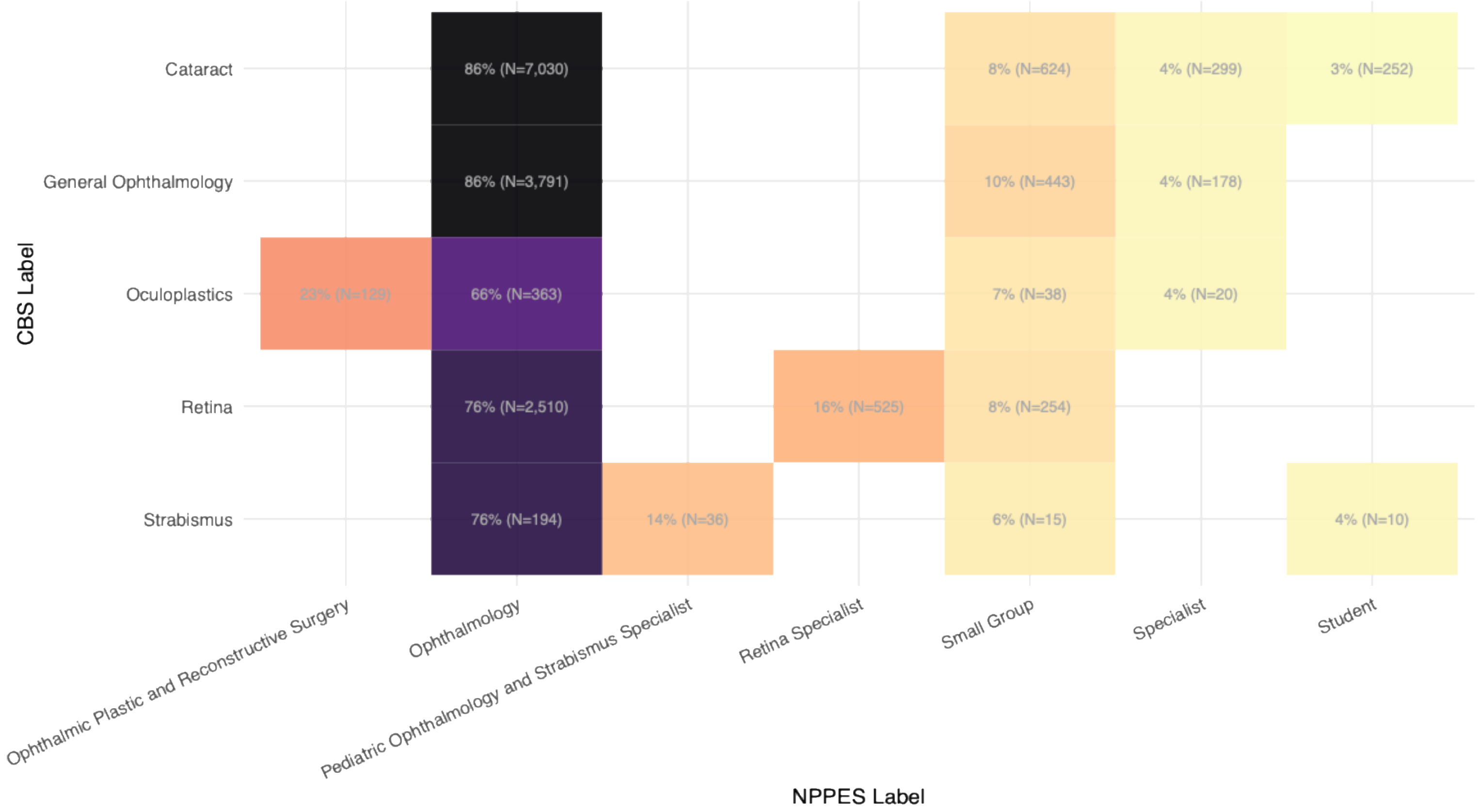

Notes: This figure shows the proportion of physicians with a specific cluster-based subspecialty label as a fraction of all physicians with a respective National Plan and Provider Enumeration System (NPPES) label. The x-axis depicts NPPES labels for ophthalmology in 2021, while the y-axis includes all distinct cluster-based subspecialty labels in 2021. Blank cells do not include any physicians.

© 2025 Karadakic R, et al. *JAMA Health Forum*.

Figure M7: Orthopedic Surgery 2021 – NPPES versus Cluster-Based Subspecialty Labels

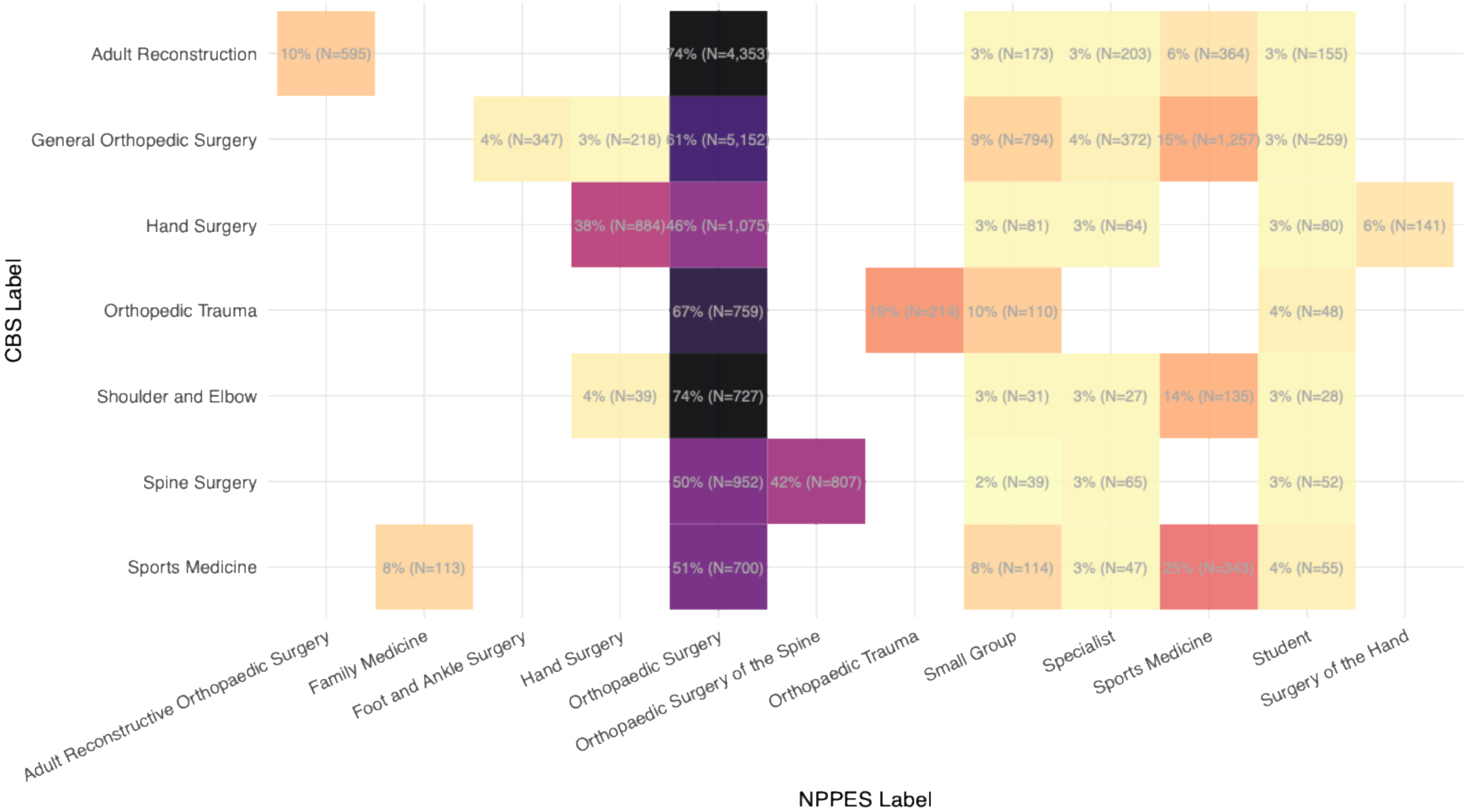

Notes: This figure shows the proportion of physicians with a specific cluster-based subspecialty label as a fraction of all physicians with a respective National Plan and Provider Enumeration System (NPPES) label. The x-axis depicts NPPES labels for orthopedic surgery in 2021, while the y-axis includes all distinct cluster-based subspecialty labels in 2021. Blank cells do not include any physicians.

Figure M8: Otolaryngology 2021 – NPPES versus Cluster-Based Subspecialty Labels

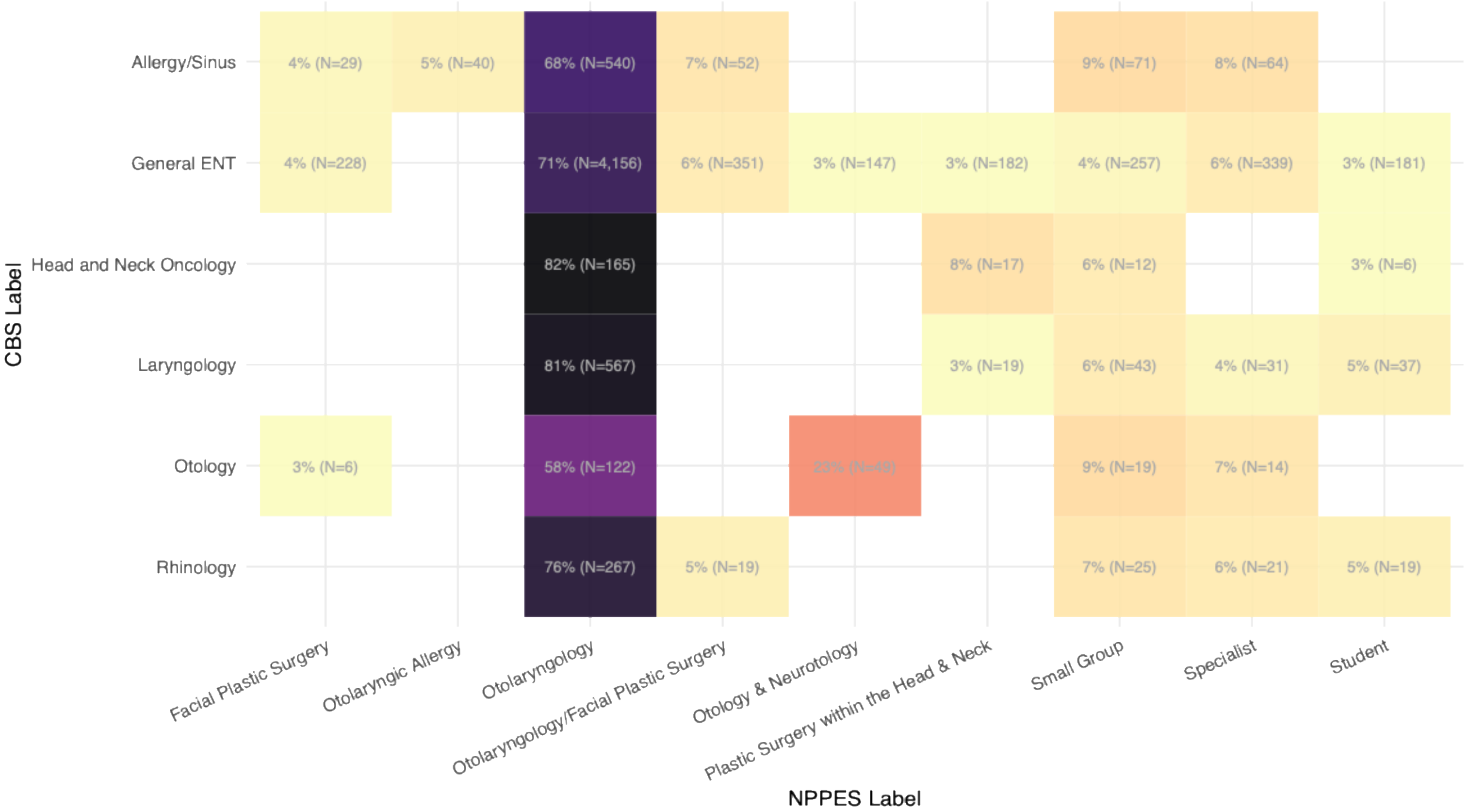

Notes: This figure shows the proportion of physicians with a specific cluster-based subspecialty label as a fraction of all physicians with a respective National Plan and Provider Enumeration System (NPPES) label. The x-axis depicts NPPES labels for

© 2025 Karadakic R, et al. *JAMA Health Forum*.

otolaryngology in 2021, while the y-axis includes all distinct cluster-based subspecialty labels in 2021. Blank cells do not include any physicians.

Label Comparison using t-SNE

As a final validation, we employed a dimensionality reduction and visualization technique called t-distributed Stochastic Neighbor Embedding (t-SNE).[4] t-SNE reduces high-dimensional data into a two-dimensional space, allowing us to plot each physician separately for each year. This technique is based on the premise that observations with similar underlying features will be positioned closer together, while those with unrelated features will be further apart. While t-SNE provides valuable visual insights, it has known limitations, including sensitivity to hyperparameters such as perplexity.[5] To ensure robustness, we tested our visualizations across different perplexity levels and found that the visual distinction between cluster-based subspecialty labels and Medicare-determined subspecialty labels remained consistent. All t-SNE figures were constructed using a perplexity of 50.

By combining the t-SNE dimensions with cluster-based subspecialty labels and Medicare-determined subspecialty labels, we can visually inspect the correspondence between labels and the two-dimensional representation of physicians. It is important to emphasize that clustering (including the assignment of subspecialty labels) and t-SNE dimensionality reduction are independent methods. Therefore, any overlap between t-SNE groupings and our subspecialty labels would only occur if the underlying dimensionality reduction reflects clinically relevant groupings that are also captured in physician specialty labels. This visual inspection helps confirm that our algorithm accurately identifies meaningful clinical distinctions among physicians. Given the limitations of t-SNE, we do not interpret the specific geometry of clusters or distances between clusters as meaningful, but rather the separation of clusters from one another.

Figure M9: t-SNE General Surgery 2021 – Medicare-Determined versus Cluster-Based Subspecialty Labels

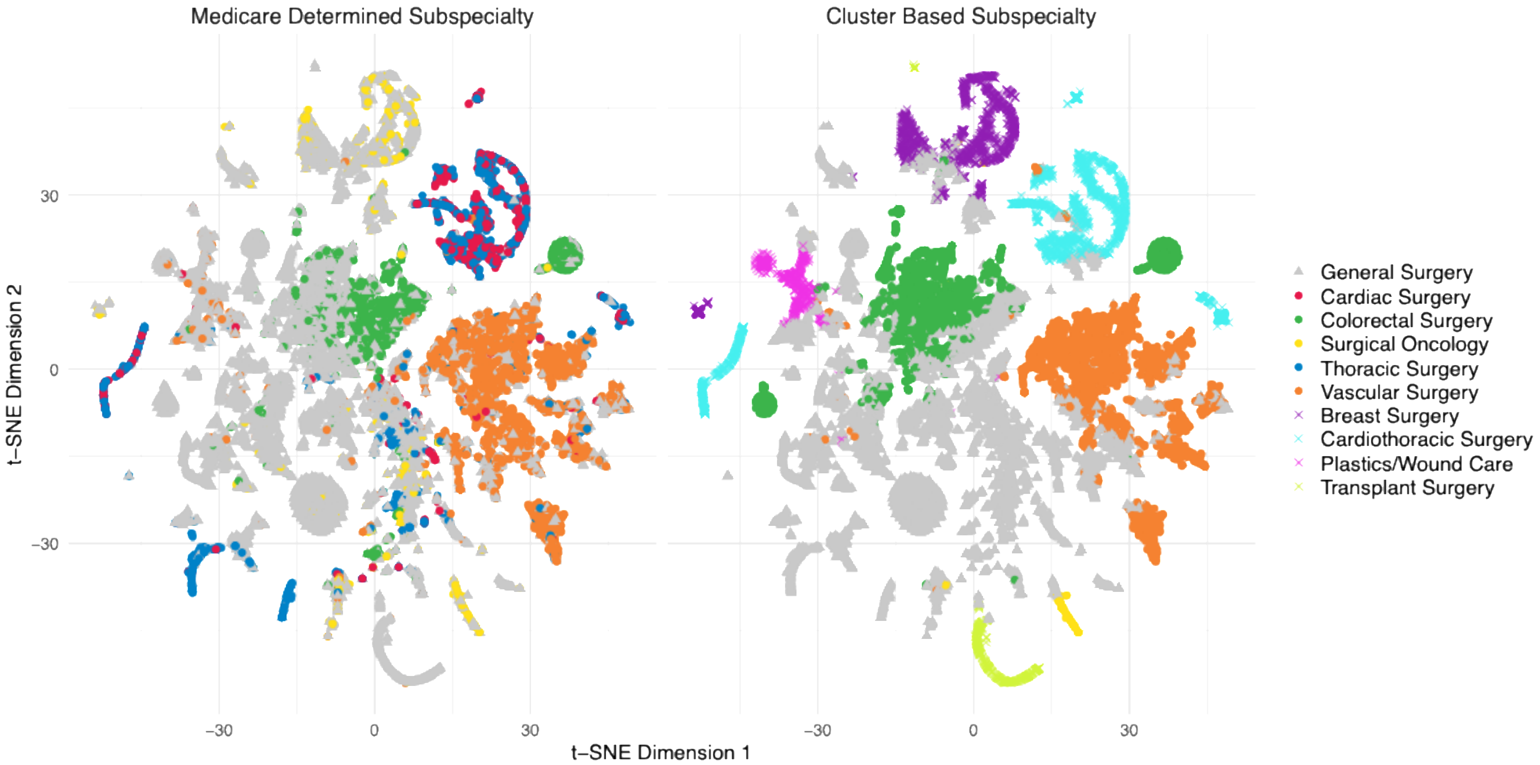

Notes: This figure presents t-SNE visualizations of physicians' procedure shares in general surgery. Different colors and shapes represent the assigned subspecialty labels, obtained either from our Cluster-Based Subspecialty Labels (CBS) on the right or from the Medicare-Determined Subspecialty Labels (MDS) on left. The general category of each specialty is indicated in grey using a triangle marker.

© 2025 Karadakic R, et al. *JAMA Health Forum*.

Figure M10: t-SNE Neurosurgery 2021 – Medicare-Determined versus Cluster-Based Subspecialty Labels

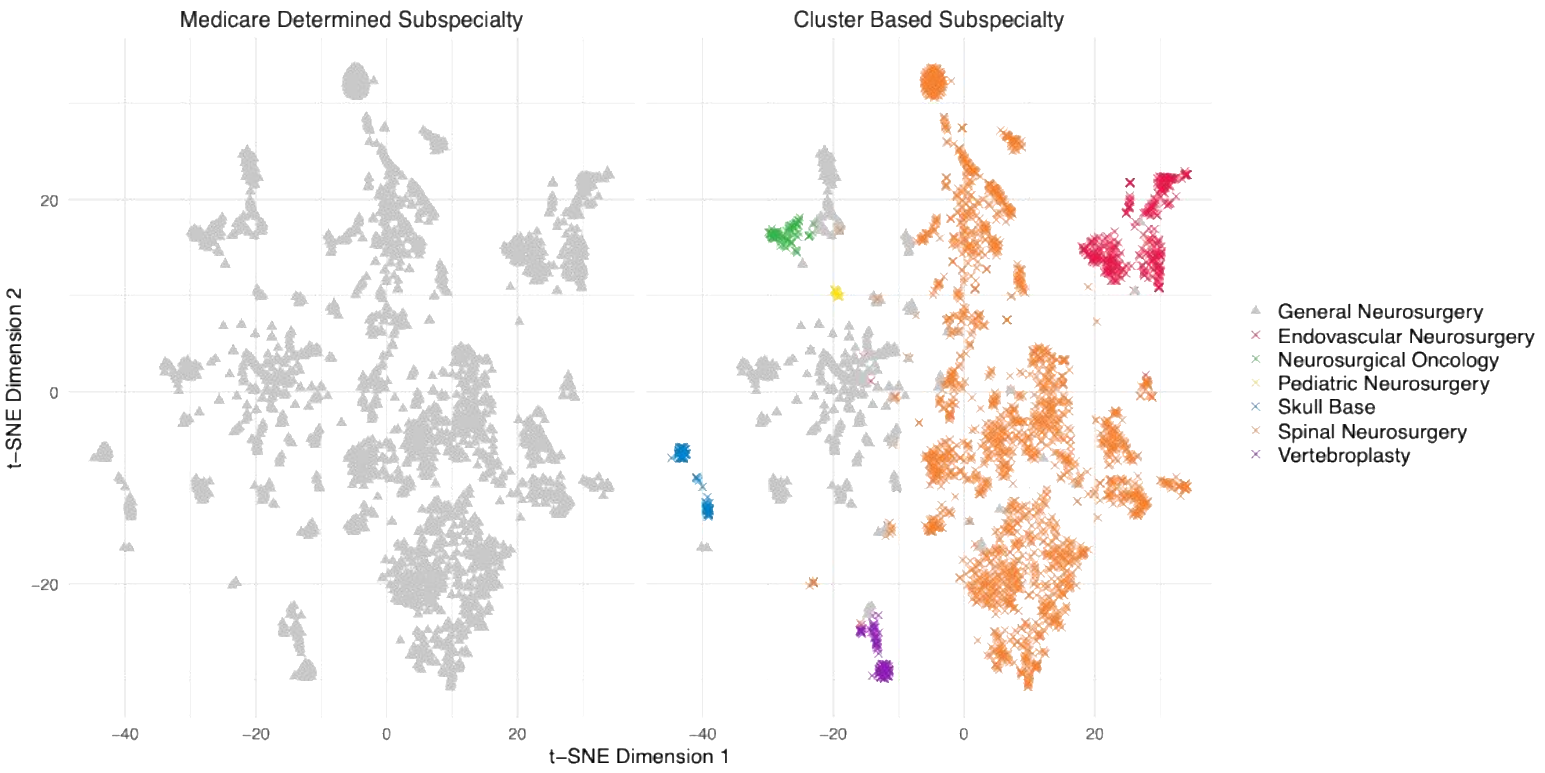

Notes: This figure presents t-SNE visualizations of physicians' procedure shares in neurosurgery. Different colors and shapes represent the assigned subspecialty labels, obtained either from our Cluster-Based Subspecialty Labels (CBS) on the right or from the Medicare-Determined Subspecialty Labels (MDS) on left. The general category of each specialty is indicated in grey using a triangle marker.

Figure M11: t-SNE Ophthalmology 2021 – Medicare-Determined versus Cluster-Based Subspecialty Labels

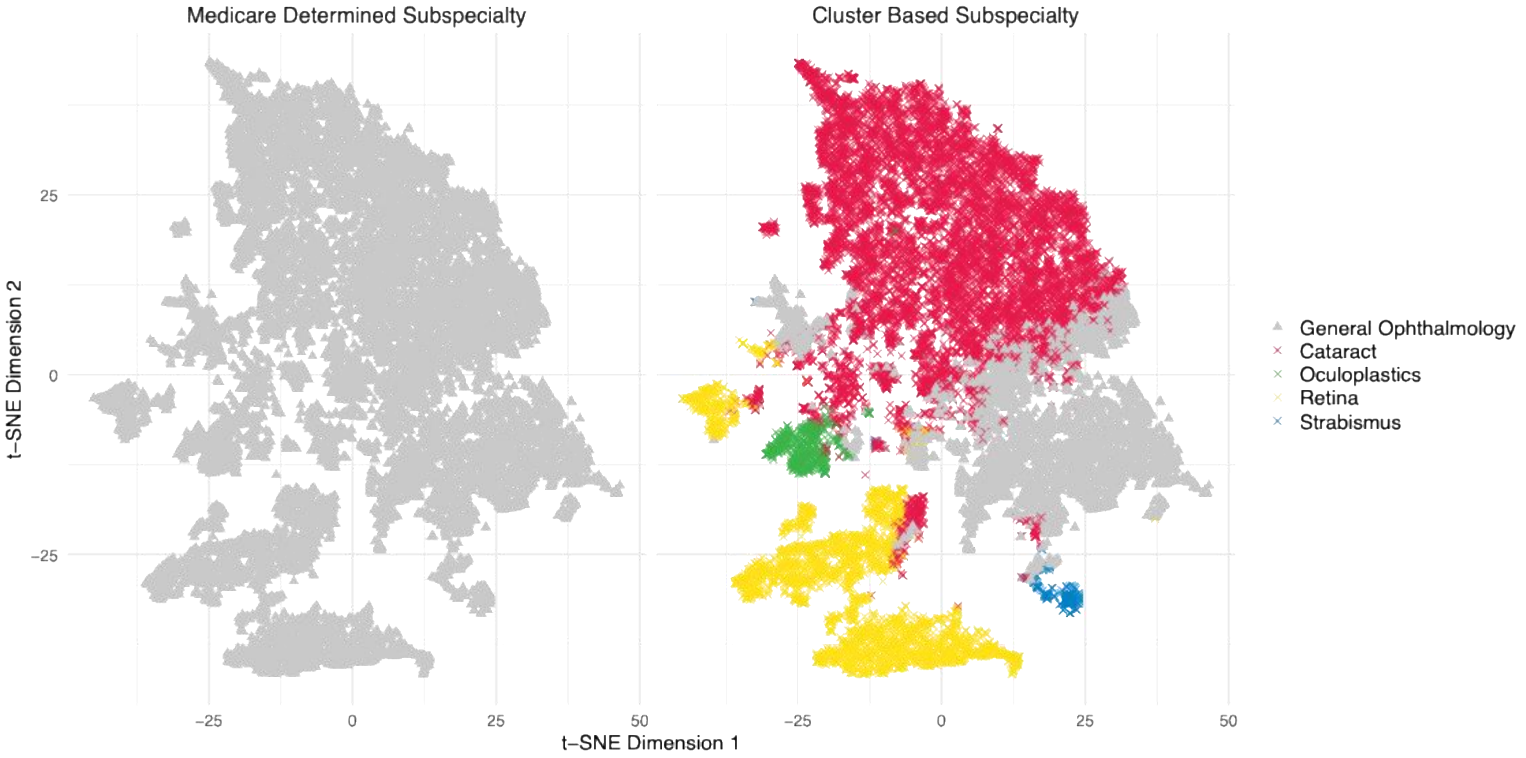

Notes: This figure presents t-SNE visualizations of physicians' procedure shares in ophthalmology. Different colors and shapes represent the assigned subspecialty labels, obtained either from our Cluster-Based Subspecialty Labels (CBS) on the right or from the Medicare-Determined Subspecialty Labels (MDS) on left. The general category of each specialty is indicated in grey using a triangle marker.

© 2025 Karadakic R, et al. *JAMA Health Forum*.

Figure M12: t-SNE Orthopedic Surgery 2021 – Medicare-Determined versus Cluster-Based Subspecialty Labels

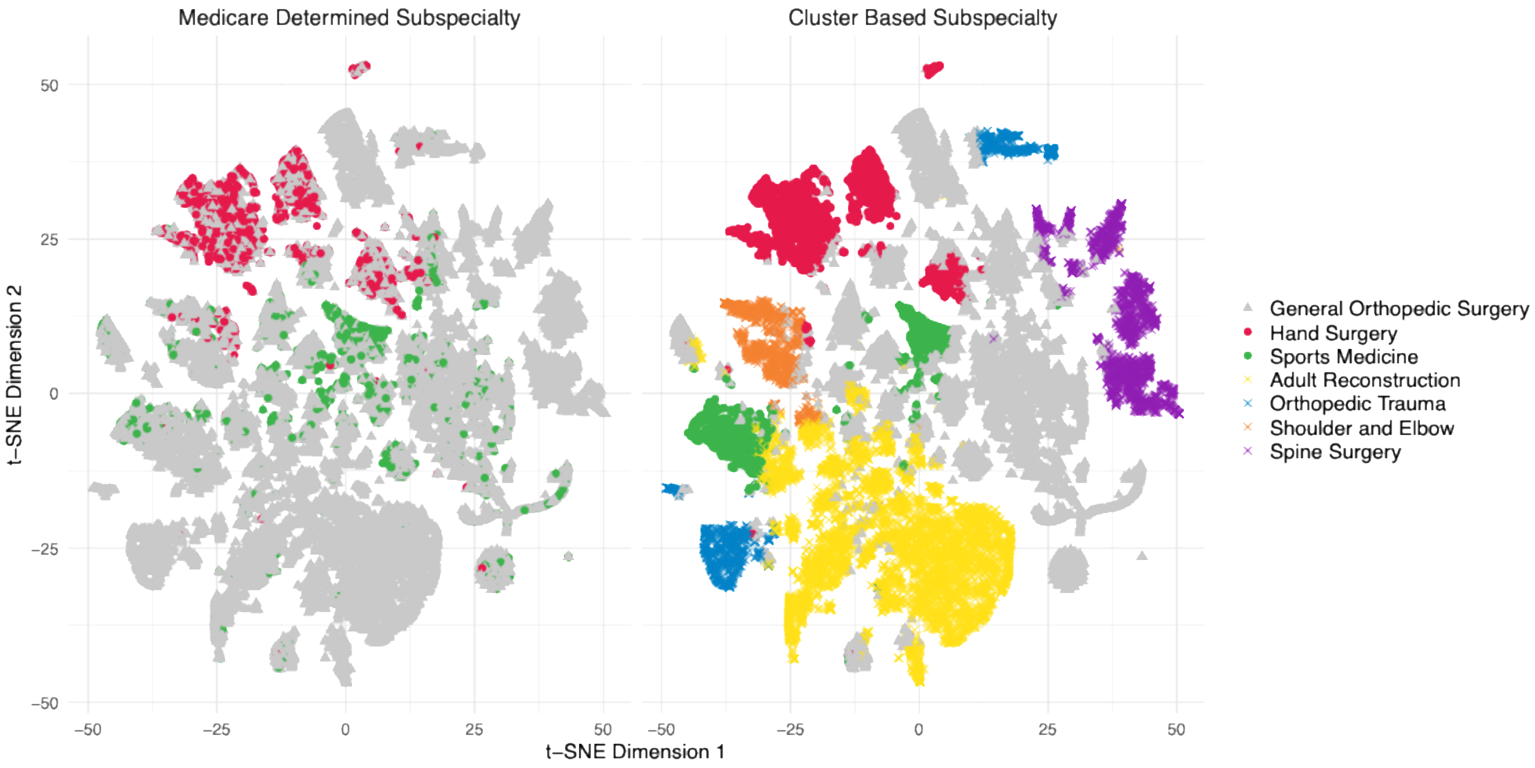

Notes: This figure presents t-SNE visualizations of physicians' procedure shares in orthopedic surgery. Different colors and shapes represent the assigned subspecialty labels, obtained either from our Cluster-Based Subspecialty Labels (CBS) on the right or from the Medicare-Determined Subspecialty Labels (MDS) on left. The general category of each specialty is indicated in grey using a triangle marker.

Figure M13: t-SNE Otolaryngology 2021 – MDS versus CBS labels

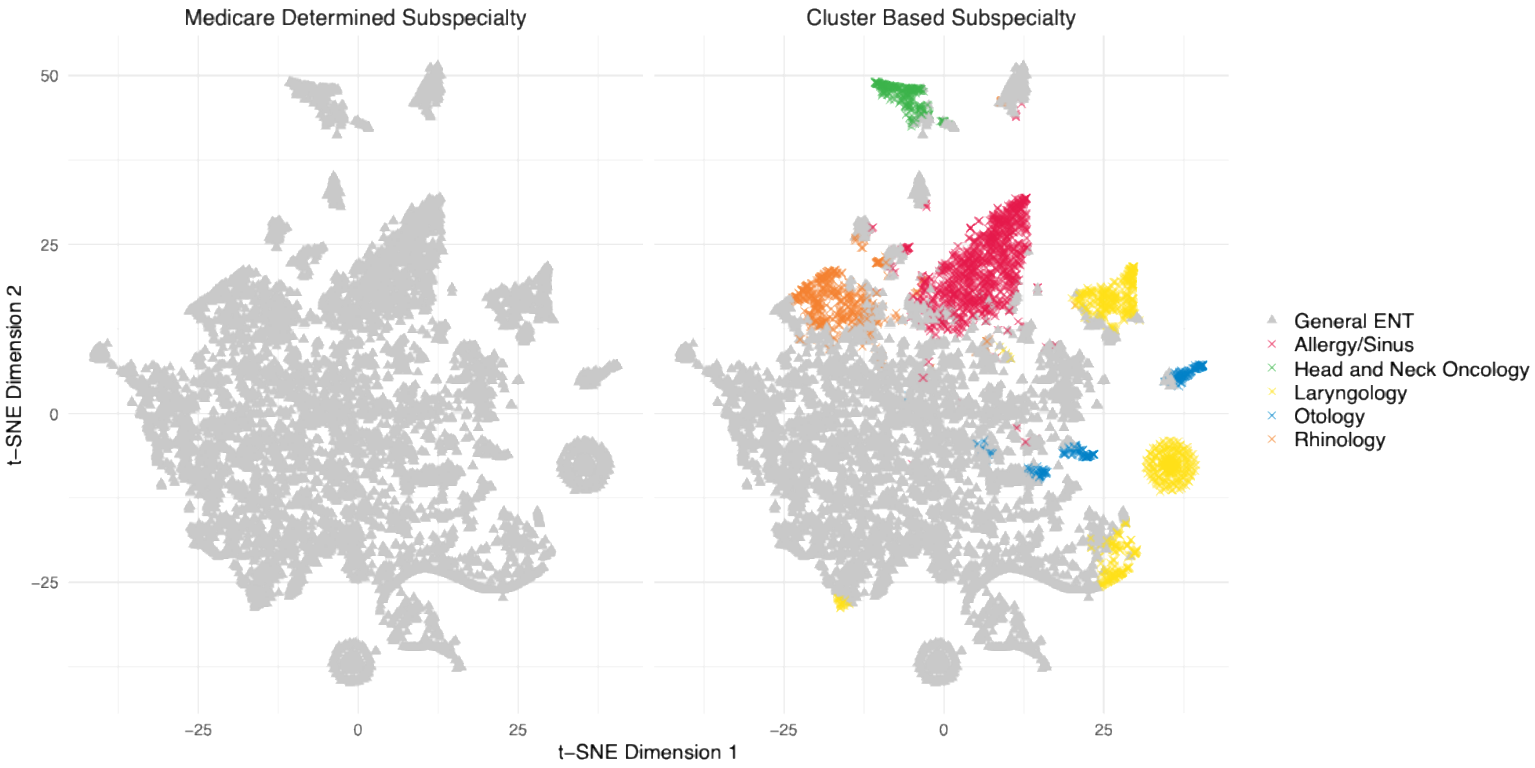

Notes: This figure presents t-SNE visualizations of physicians' procedure shares in otolaryngology. Different colors and shapes represent the assigned subspecialty labels, obtained either from our Cluster-Based Subspecialty Labels (CBS) on the right or from the Medicare-Determined Subspecialty Labels (MDS) on left. The general category of each specialty is indicated in grey using a triangle marker.

© 2025 Karadakic R, et al. *JAMA Health Forum*.

Regression Model: Surgical Generalist's Practice Scope

The association between subspecialization and the practice scope of generalist surgeons was evaluated using regression models specified as follows:

$$\log(y_{i,h,t}) = \alpha + \beta_1 \log(Subspec_{h,s,t}) + \beta_2 X_{h,s,t} + \gamma_s + \tau_t + \psi_r + \varepsilon_{i,h,t}$$

In this equation $\log(y_{i,h,t})$ represented the logarithm of the outcome y (unique procedures performed or procedure HHI) for physician i in HRR h, calendar year t. The independent variable $Subspec_{h,s,t}$ denoted the number of subspecialists per 100,000 fee-for-service enrollees in the respective HRR and year and specialty s. The vector $X_{h,s,t}$ controlled for the number of generalists per fee-for-service population and incorporates a variable accounting for the fee-for-service population size at the HRR level. The model included fixed effects for specialty γ_s, calendar year τ_t, and the state of the physician's most frequent practice location ψ_r. The coefficient β_1 captured the percentage change in the outcome associated with a one percentage point increase in the number of subspecialists per 100,000 fee-for-service enrollees.

© 2025 Karadakic R, et al. *JAMA Health Forum*.

Supplemental Figures

Figure S1: t-SNE Representation of Physicians by Specialty 2021

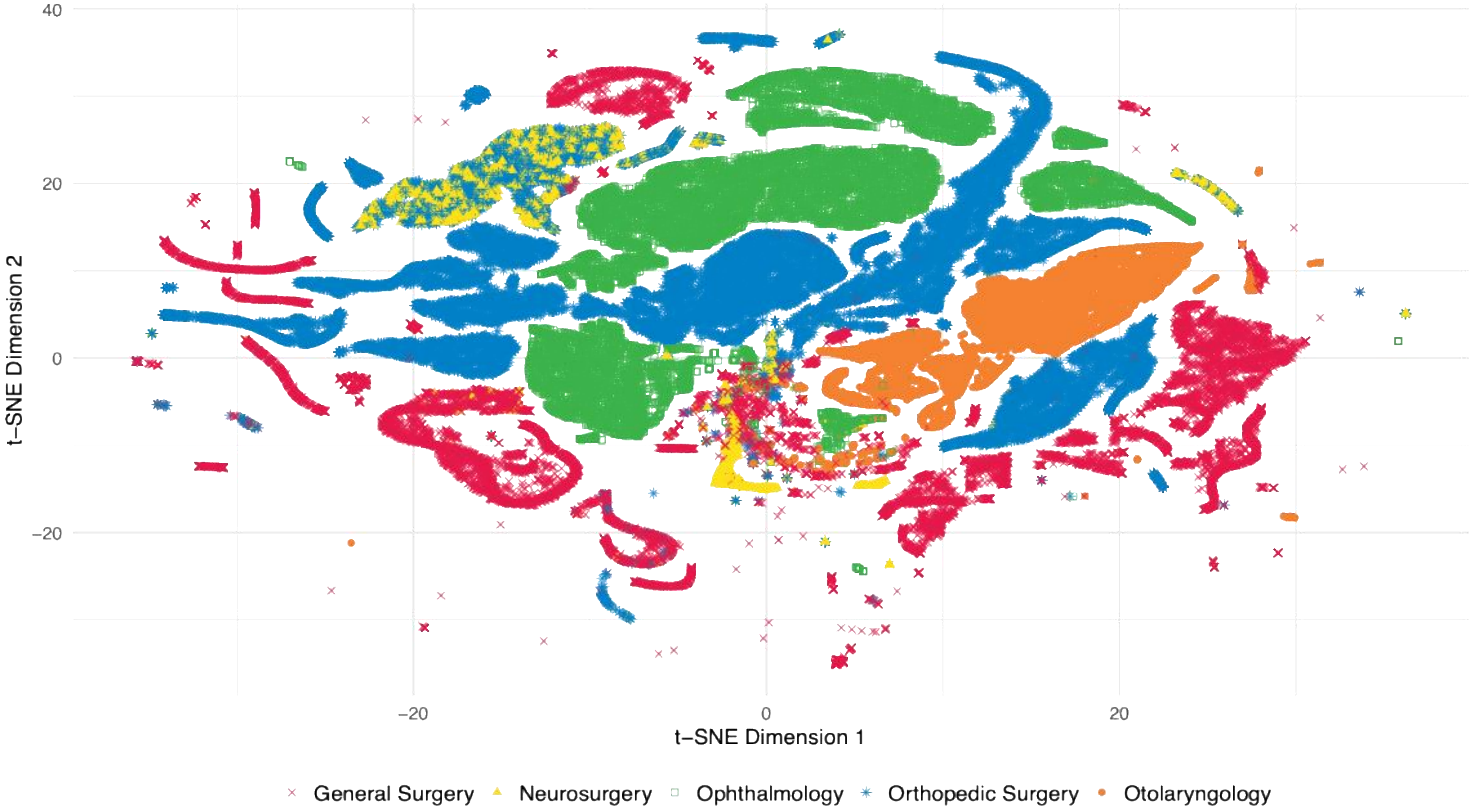

Notes: This figure presents t-SNE visualizations of physicians' procedure shares for all physicians in our main sample in the year 2021. All five surgical specialty groups (general surgery, neurosurgery, ophthalmology, orthopedic surgery, otolaryngology) are included. Each marker represents a single physician. Colors and markers indicate the respective specialty as defined using the Medicare healthcare provider taxonomy.

© 2025 Karadakic R, et al. *JAMA Health Forum*.

Figure S2: Medicare Fee-For-Service Population Growth

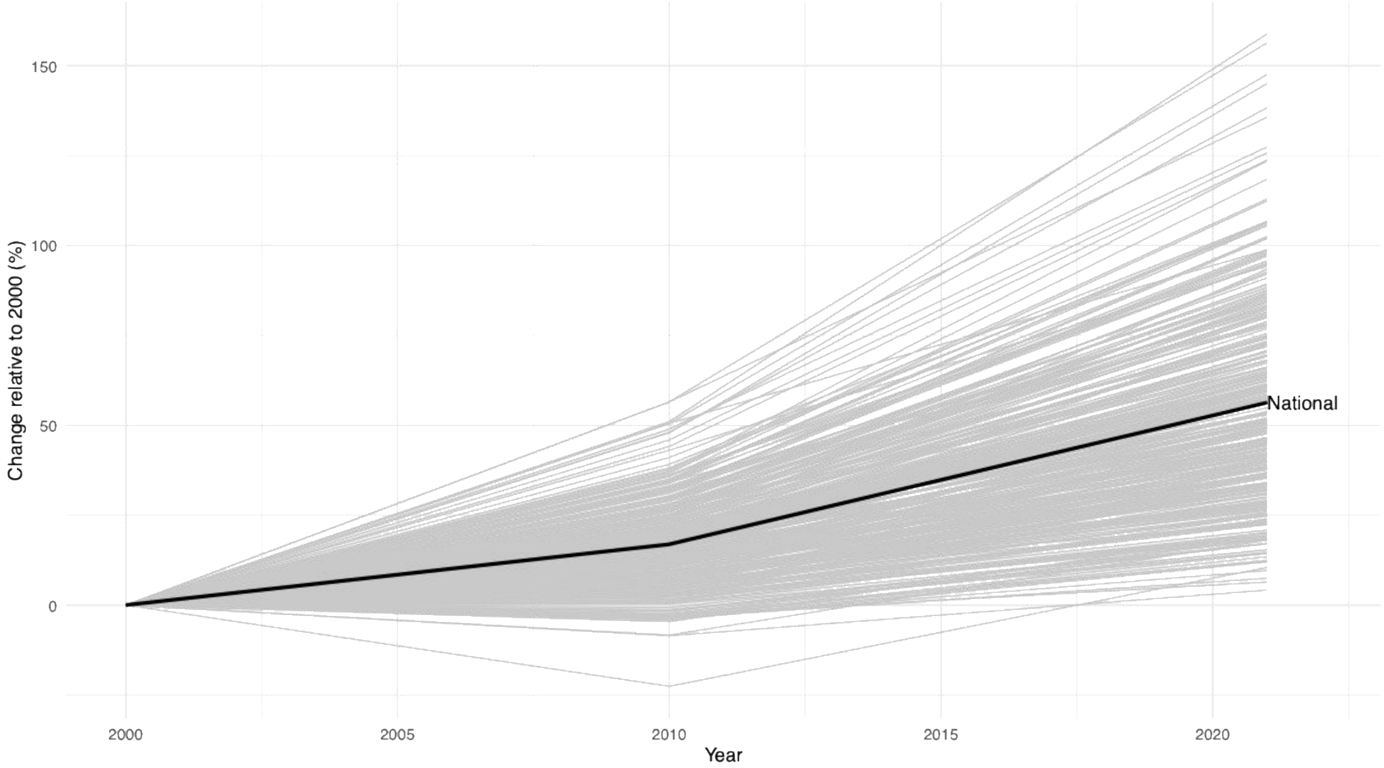

Notes: This figure shows the growth in the fee-for-service Medicare population from 2000 to 2021. The national trend line is highlighted in black and thin grey lines are trend lines for individual HRRs. The y-axis represents the change in the fee-for-service population relative to the base year (2000) in percent.

© 2025 Karadakic R, et al. *JAMA Health Forum*.

Figure S3: Share of Subspecialists by Graduation Year in 2021

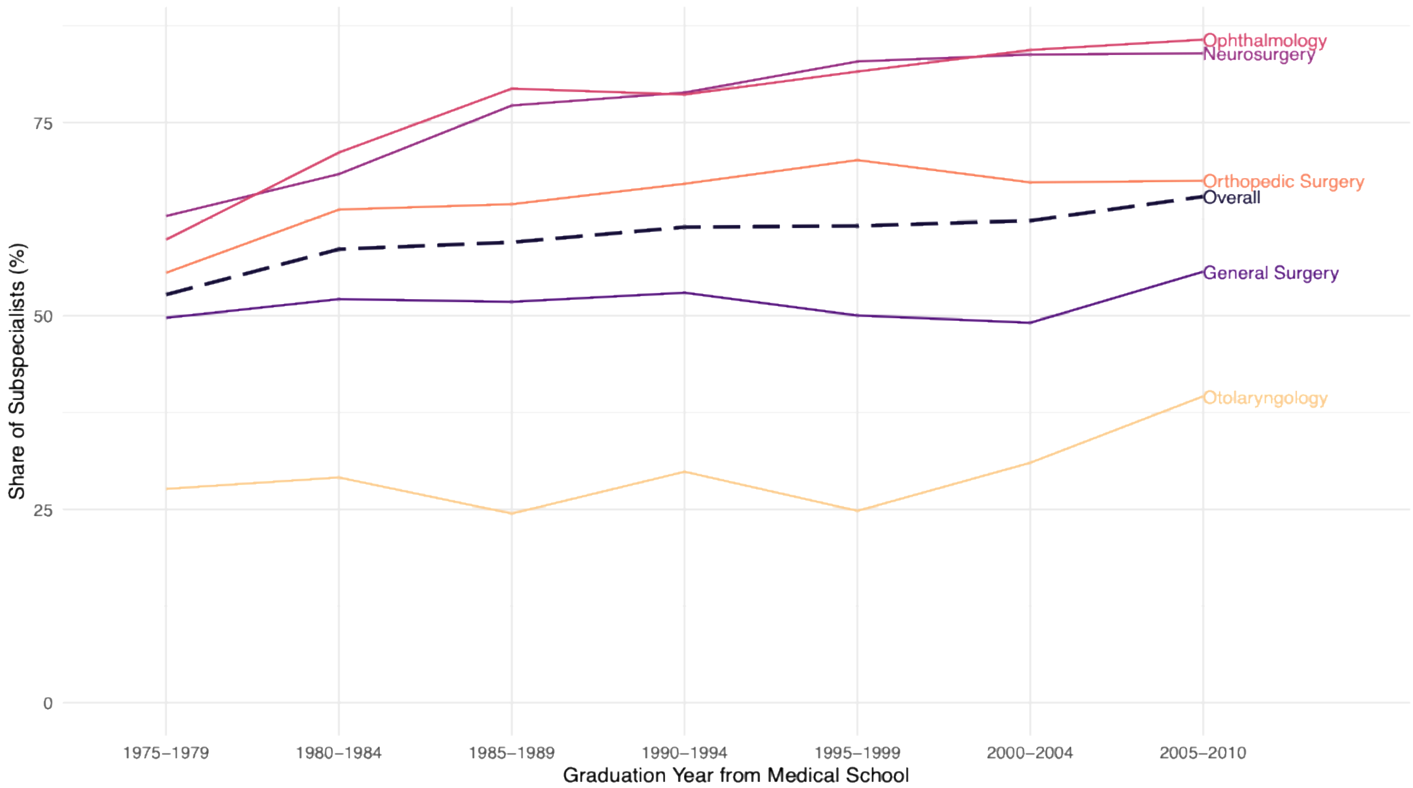

Notes: This figure presents the share of physicians classified as subspecialists according to our cluster-based subspecialty labels, categorized by their respective graduation years from medical school. Each dot represents the average number of subspecialists by graduation year in five-year bins (last bin includes six years). Each line indicates the subspecialization share for a different specialty indicated by different colors and markers; the Overall group consists of the subspecialization share for the entire sample across all specialties.

© 2025 Karadakic R, et al. *JAMA Health Forum*.

Figure S4: Geographic Variation of Physicians across HRRs, Specialties and Time

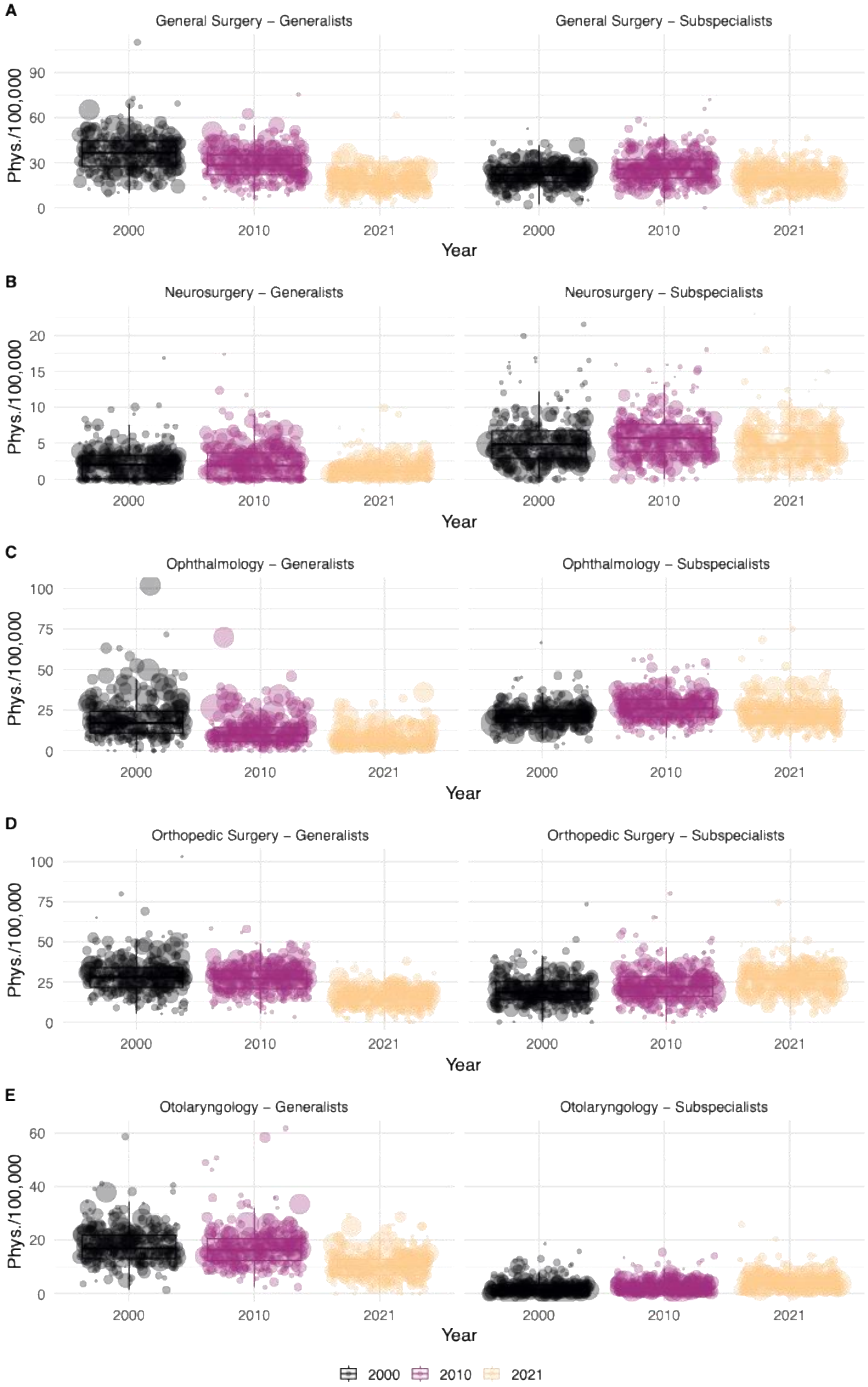

Notes: This figure presents boxplots and jitter plots of generalist and subspecialist physicians per 100,000 fee-for-service Medicare enrollees across hospital referral regions (HRRs) and specialties for the years 2000, 2010, and 2021. All five surgical specialty groups (general surgery, neurosurgery, ophthalmology, orthopedic surgery, otolaryngology) are included. The left panel shows the share of generalist surgeons of the respective specialty per 100,000 within an HRR and the right panel shows the number of subspecialists per 100,000 fee-for-service enrollees. ach bubble represents an HRR. The bubble size is proportional to the size of HRRs in 2000.

© 2025 Karadakic R, et al. *JAMA Health Forum*.

Figure S5: Geographic Variation of Physicians across HRRs over Time with Constant Fee-For-Service Population

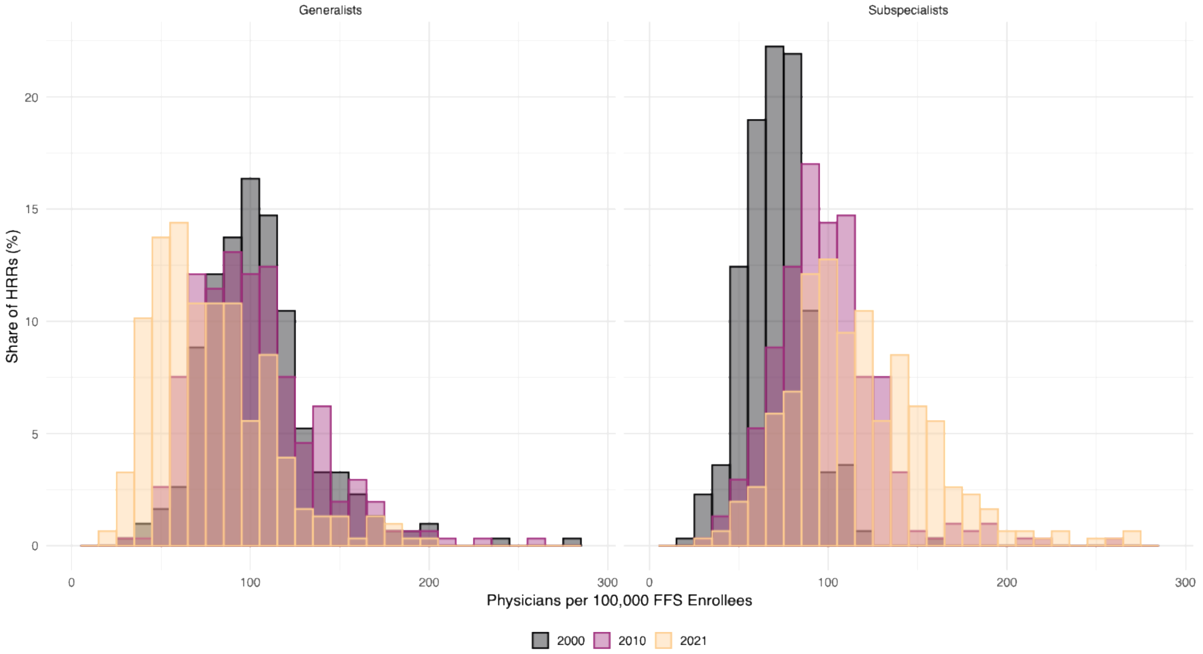

Notes: This figure presents histograms of generalist and subspecialist physicians per 100,000 fee-for-service Medicare enrollees across hospital referral regions (HRRs) for the years 2000, 2010, and 2021 while holding the Medicare population in each HRR constant at the year 2000 level. The left panel shows the share of generalists per 100,000 within an HRR and the right panel shows the number of subspecialists per 100,000 fee-for-service enrollees. All five surgical specialty groups (general surgery, neurosurgery, ophthalmology, orthopedic surgery, otolaryngology) are included. Surgical generalists and subspecialists were classified using cluster-based subspecialty labels.

© 2025 Karadakic R, et al. *JAMA Health Forum*.

Figure S6: Geographic Variation of Physicians across HRRs over Time with Constant Fee-For-Service Population (Medicare-Determined Subspecialty Labels)

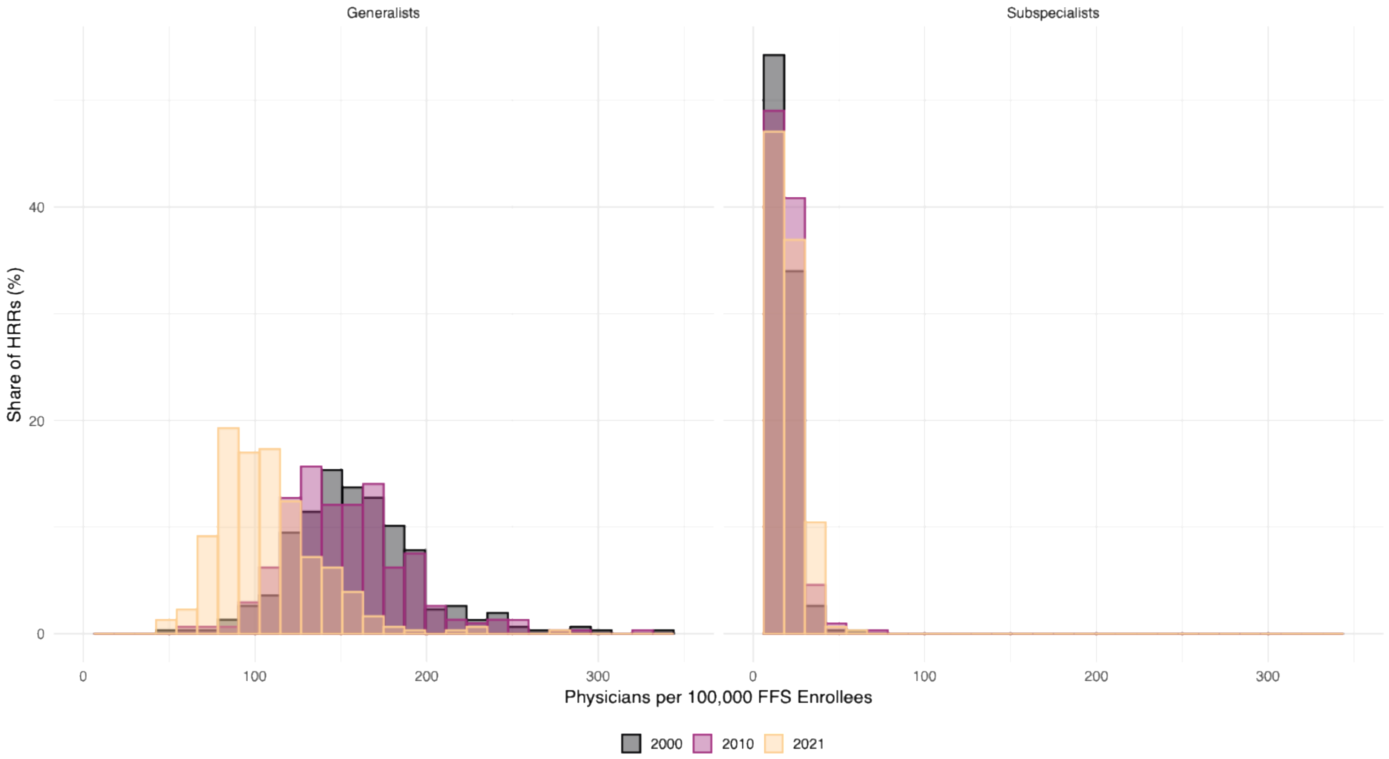

<u>Notes:</u> This figure presents histograms of generalist and subspecialist physicians per 100,000 fee-for-service Medicare enrollees across hospital referral regions (HRRs) for the years 2000, 2010, and 2021 while holding the Medicare population in each HRR constant at the year 2000 level. The left panel shows the share of generalists per 100,000 within an HRR and the right panel shows the number of subspecialists per 100,000 fee-for-service enrollees. All five surgical specialty groups (general surgery, neurosurgery, ophthalmology, orthopedic surgery, otolaryngology) are included. Surgical generalists and subspecialists were classified using Medicare-determined subspecialty labels.

© 2025 Karadakic R, et al. *JAMA Health Forum*.

Figure S7: Association between Surgical Generalists/Subspecialists and Fee-for-Service Population within HRRs from 2000, 2010 and 2021

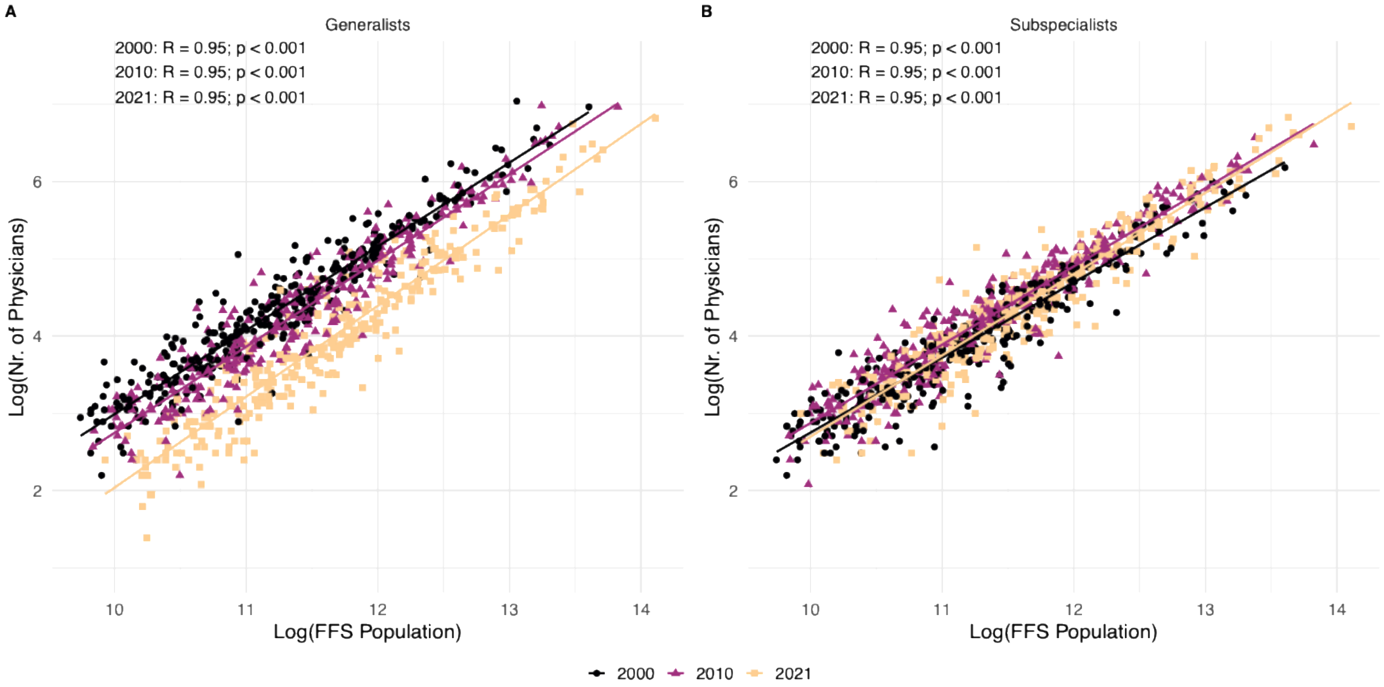

Notes*:* This figure plots the association between the number of surgical generalists and subspecialists within hospital referral regions (HRRs) for the years 2000, 2010, and 2021. In panel A (left), the y-axis represents the natural logarithm of the number of surgical generalists, while the x-axis represents the natural logarithm of the fee-for-service Medicare population within an HRR. In panel B (right), the y-axis represents the natural logarithm of the number of subspecialists, while the x-axis represents the natural logarithm of the fee-for-service Medicare population within an HRR. Both panels include Pearson correlation coefficients for each year, with corresponding p-values displayed in the top-left corner. Different markers indicate the respective years, and fitted lines represent best-fit estimates from a linear model. All five surgical specialties (general surgery, neurosurgery, ophthalmology, orthopedic surgery, and otolaryngology) are included.

© 2025 Karadakic R, et al. *JAMA Health Forum*.

Figure S8: Geographic Variation of Subspecialists across 50 Largest HRRs

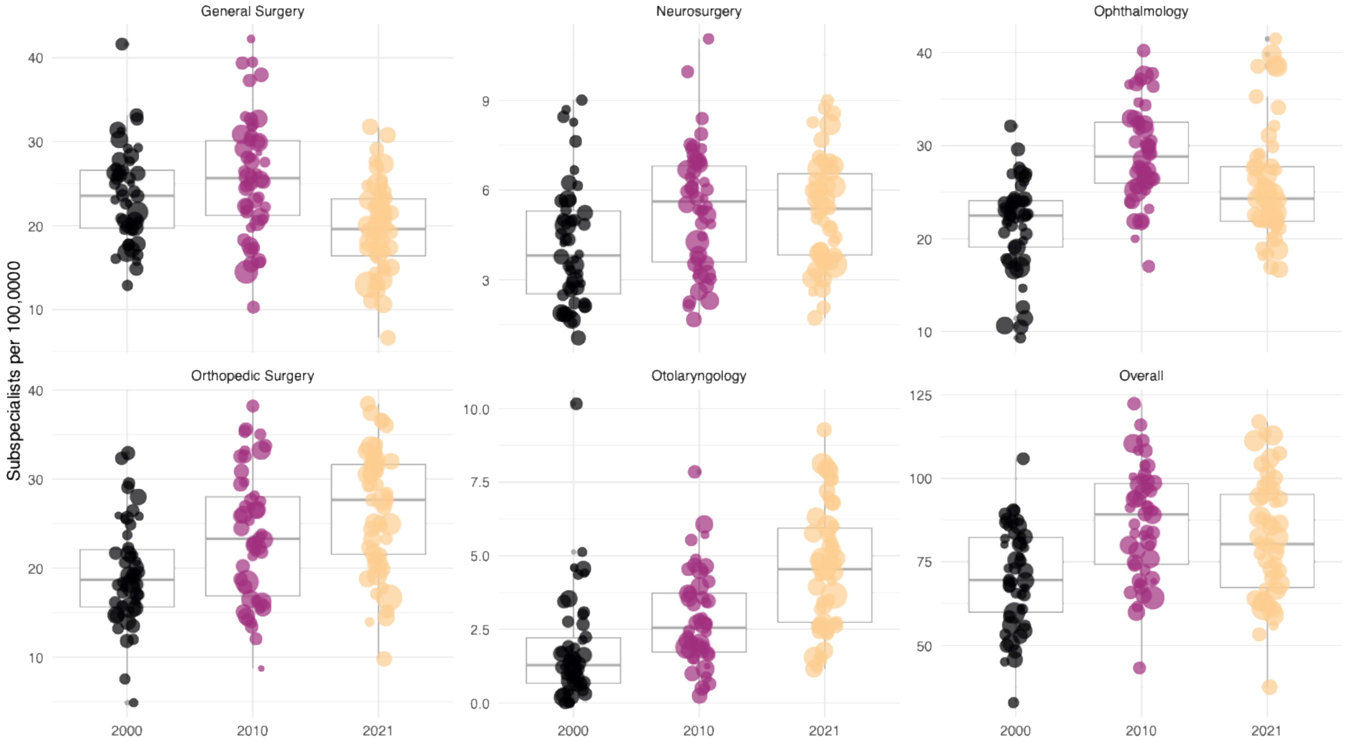

Notes: This figure presents boxplots of the number of subspecialist physicians per 100,000 fee-for-service Medicare enrollees across hospital referral regions (HRRs) for the years 2000, 2010, and 2021. Each panel shows subspecialists per beneficiaries for the 50 largest HRRs (2000 level) for different specialties and across all specialties combined. All five surgical specialty groups (general surgery, neurosurgery, ophthalmology, orthopedic surgery, otolaryngology) are included.

© 2025 Karadakic R, et al. *JAMA Health Forum*.

Figure S9: Change in Subspecialist Share from 2000 to 2021 relative to the Number of Surgeons per 100,000 FFS Enrollees across HRRs

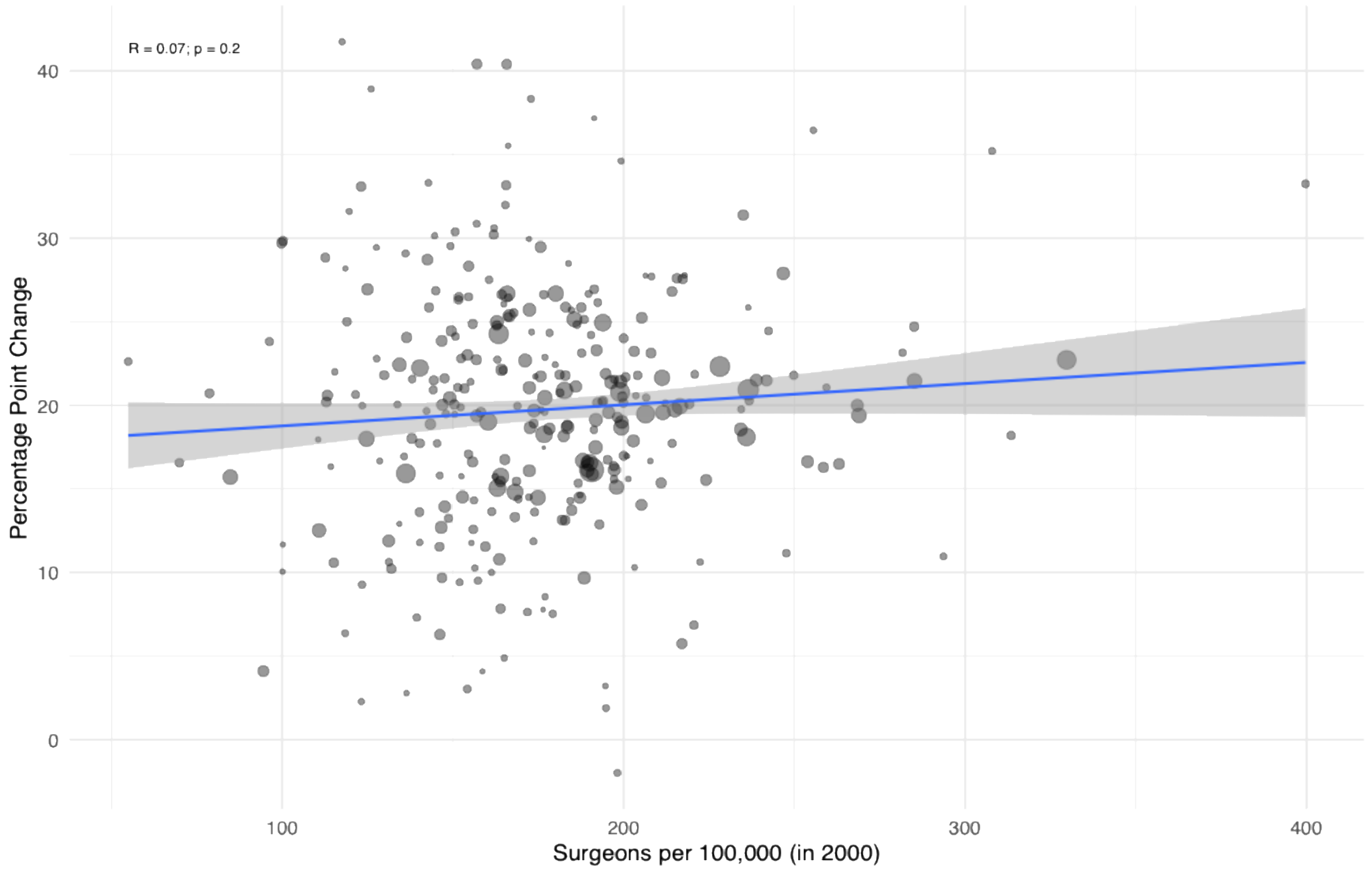

Notes*:* This figure presents the relationship between the number of surgeons, defined as all physicians in the five specialties in our main sample, per 100,000 fee-for-service Medicare enrollees (x-axis) and the percentage point change in the share of subspecialist surgeons from 2000 to 2021 (y-axis). All five surgical specialty groups (general surgery, neurosurgery, ophthalmology, orthopedic surgery, otolaryngology) are included.

© 2025 Karadakic R, et al. *JAMA Health Forum*.

Figure S10: Change in Subspecialist Share relative to the Number of Surgeons per 100,000 FFS Enrollees, by Specialty across HRRs

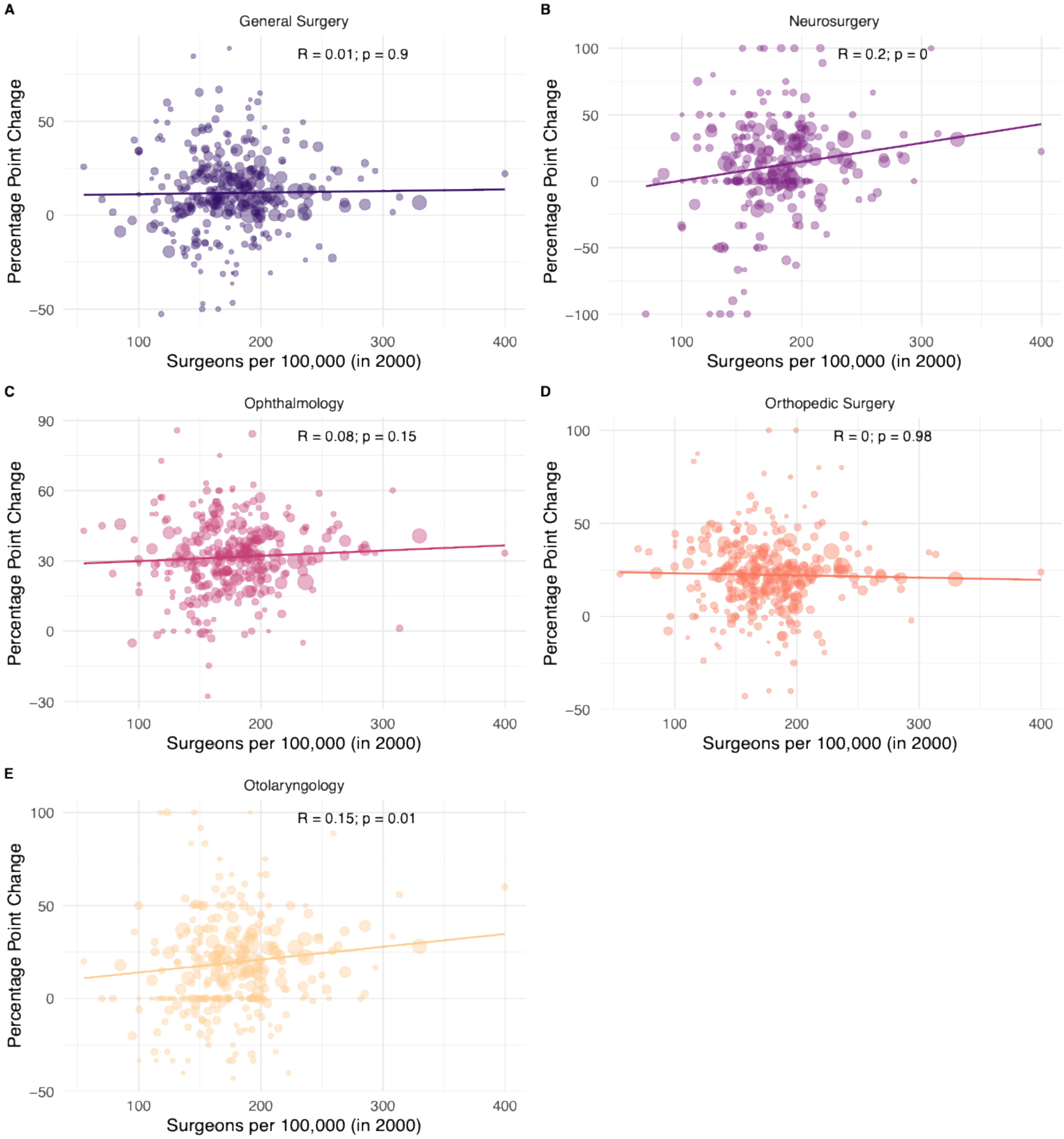

Notes: This figure presents the relationship between the number of surgeons, defined as all physicians in the five specialties in our main sample, per 100,000 fee-for-service Medicare enrollees (x-axis) and the percentage point change in the share of subspecialist surgeons from 2000 to 2021 within each of the specialties (y-axis). Each dot represents an individual HRR. Different panels show the relationship between the two variables for the respective specialties (general surgery, neurosurgery, ophthalmology, orthopedic surgery, otolaryngology).

© 2025 Karadakic R, et al. *JAMA Health Forum*.

Supplemental Tables

Table S1: Specialty Codes Defining Inclusion of Physicians in Main Sample

CMS Description	CMS Code	Specialty Group
General Surgery	02	General Surgery
Colorectal Surgery	28	General Surgery
Thoracic Surgery	77	General Surgery
Vascular Surgery	77	General Surgery
Cardiac Surgery	78	General Surgery
Surgical Oncology	91	General Surgery
Neurosurgery	14	Neurosurgery
Ophthalmology	18	Ophthalmology
Orthopedic Surgery	20	Orthopedic Surgery
Sports Medicine	23	Orthopedic Surgery
Hand Surgery	40	Orthopedic Surgery
Otolaryngology	04	Otolaryngology

Notes: This table includes the CMS specialty codes defining each of the five main specialties comprising the physicians in our main sample. Column 1 indicates the description of the CMS specialty code, column 2 the respective CMS specialty code and column 3 the specialty to which we assigned the CMS specialty code.

© 2025 Karadakic R, et al. *JAMA Health Forum*.

Table S2: Summary Statistics of Main Sample of Physicians by Specialty and Year

	2000	2010	2021
**General Surgery**			
Number of Physicians	19,630	21,893	19,665
Total Annual Services	726 (1,079)	895 (1,214)	733 (5,923)
Total Annual Revenue ($)	65,323 (74,622)	80,840 (128,499)	72,113 (183,360)
Unique Procedures	11.6 (7.3)	13.8 (10.0)	10.2 (6.5)
Revenue Share (%)	8.2 (11.5)	7.2 (10.8)	9.8 (12.6)
**Neurosurgery**			
Number of Physicians	2,217	3,155	3,247
Total Annual Services	500 (515)	769 (1,435)	603 (1,262)
Total Annual Revenue ($)	55,751 (52,190)	93,338 (134,935)	82,276 (91,910)
Unique Procedures	9.4 (5.1)	14.1 (8.2)	11.1 (6.2)
Revenue Share (%)	10.2 (12.2)	7.1 (9.6)	9.0 (10.6)
**Ophthalmology**			
Number of Physicians	14,919	16,555	16,781
Total Annual Services	2,168 (1,861)	3,809 (4,154)	3,139 (3,971)
Total Annual Revenue ($)	173,870 (191,899)	293,552 (467,068)	364,213 (696,896)
Unique Procedures	12.6 (5.9)	16.7 (8.4)	13.4 (6.1)
Revenue Share (%)	7.7 (12.5)	6.0 (10.9)	7.5 (12.5)
**Orthopedic Surgery**			
Number of Physicians	16,355	19,989	22,025
Total Annual Services	1,171 (1,215)	1,694 (1,757)	2,316 (5,399)
Total Annual Revenue ($)	62,852 (67,867)	102,088 (109,083)	99,516 (114,676)
Unique Procedures	15.9 (8.4)	18.1 (10.0)	14.0 (7.4)
Revenue Share (%)	6.0 (9.0)	5.5 (8.7)	7.1 (10.1)
**Otolaryngology**			
Number of Physicians	6,933	7,977	8,117
Total Annual Services	1,156 (1,020)	1,423 (1,507)	1,483 (2,544)
Total Annual Revenue ($)	46,057 (42,507)	72,527 (81,396)	75,526 (95,467)
Unique Procedures	12.0 (6.1)	13.0 (7.0)	10.5 (5.2)
Revenue Share (%)	7.7 (9.3)	7.7 (10.0)	9.5 (10.9)

Notes: This table provides summary statistics for all physicians in our main sample, encompassing five different specialties for the years 2000, 2010, and 2021. Total annual services are defined as the number of services rendered by a physician for different HCPCS codes over a year. Total annual revenue is the sum of number of services for each HCPCS code multiplied with the respective average Medicare payment amount of the respective HCPCS code. Revenue shares are defined as the share of a procedure's revenue among the total revenue of a physician (See the Subspecialty Detection Method section in the supplement).

© 2025 Karadakic R, et al. *JAMA Health Forum*.

Table S3: Trends in the Number of Distinct Subspecialties

	**2000**		**2010**		**2021**	
	**CBS**	**MDS**	**CBS**	**MDS**	**CBS**	**MDS**
General Surgery	8	6	7	6	8	6
Neurosurgery	3	1	5	1	7	1
Ophthalmology	7	1	5	1	5	1
Orthopedic Surgery	4	2	7	2	7	3
Otolaryngology	2	1	4	1	6	1
**Total**	**24**	**11**	**28**	**11**	**33**	**12**

Notes: This table presents the number of unique subspecialty labels within each specialty for the years 2000, 2010 and 2021. The table separately provides the number of unique subspecialties by year as measured using our cluster-based subspecialty (CBS) labels and Medicare determined subspecialty labels (MDS).

© 2025 Karadakic R, et al. *JAMA Health Forum*.

Table S4: Share of Physicians Reclassified by Specialty and Year

**Specialty**	**2000**	**2010**	**2021**
General Surgery	19.0% (N=3,721)	24.1% (N=5,277)	23.0% (N=4,523)
Neurosurgery	66.4% (N=1,472)	65.8% (N=2,077)	77.3% (N=2,511)
Ophthalmology	47.0% (N=7,007)	64.9% (N=10,746)	73.5% (N=12,332)
Orthopedic Surgery	38.2% (N=6,239)	41.7% (N=8,332)	53.5% (N=11,787)
Otolaryngology	9.3% (N=646)	13.1% (N=1,041)	27.8% (N=2,263)
Total	31.8% (N=19,085)	39.5% (N=27,473)	57.84% (N=33,416)

Notes: This table provides the share and number of physicians in each specialty and year that were classified as generalists by their respective Medicare determined subspecialty label but were reclassified as subspecialists using our cluster-based subspecialty labels. Neurosurgery, Ophthalmology and Otolaryngology only have a general label across all years.

© 2025 Karadakic R, et al. *JAMA Health Forum*.

Table S5: Subspecialists and Generalists by Label Type and Year

**Specialty**	**Year**	**MDS**		**CBS**	
		**Generalists**	**Subspecialists**	**Generalists**	**Subspecialists**
General Surgery	2000	71.7% (N = 14,079)	28.3% (N = 5,551)	62.1% (N = 12,196)	37.9% (N = 7,434)
	2010	67.0% (N = 14,659)	33.0% (N = 7,234)	54.6% (N = 11,943)	45.5% (N = 9,950)
	2021	58.9% (N = 11,590)	41.1% (N = 8,075)	49.2% (N = 9,668)	50.8% (N = 9,997)
Neurosurgery	2000	100.0% (N= 2,217)	-	33.6% (N = 745)	66.4% (N = 1,472)
	2010	100.0% (N = 3,155)	-	34.2% (N = 1,078)	65.8% (N = 2,077)
	2021	100.0% (N = 3,247)	-	22.7% (N = 736)	77.3% (N = 2,511)
Ophthalmology	2000	100.0% (N=14,919)	-	53.0% (N = 7,912)	47.0% (N = 7,007)
	2010	100.0% (N = 16,555)	-	35.1% (N = 5,809)	64.9% (N = 10,746)
	2021	100.0% (N = 16,781)	-	26.5% (N = 4,449)	73.5% (N = 12,332)
Orthopedic Surgery	2000	97.4% (N = 15,926)	2.6% (N = 429)	60.6% (N = 9,903)	39.5% (N = 6,452)
	2010	95.4% (N = 19,074)	4.6% (N = 915)	55.3% (N = 11,044)	44.8% (N = 8,945)
	2021	86.9% (N = 19,131)	13.1% (N = 2,894)	38.3% (N = 8,435)	61.7% (N = 13,590)
Otolaryngology	2000	100.0% (N = 6,933)	-	90.7% (N = 6,287)	9.3% (N = 646)
	2010	100.0% (N = 7,977)	-	87.0% (N = 6,936)	13.0% (N = 1,041)
	2021	100.0% (N = 8,117)	-	72.1% (N = 5,854)	27.9% (N = 2,263)
Total	2000	90.0% (N = 54,074)	10.0% (N = 5,980)	61.7% (N = 37,043)	38.3% (N = 23,011)
	2010	88.3% (N = 61,420)	11.7% (N = 8,149)	52.9% (N = 36,810)	47.1% (N = 32,759)
	2021	84.3% (N = 58,866)	15.7% (N = 10,969)	41.7% (N = 29,142)	58.3% (N = 40,693)

Notes: This table provides the share and number of physicians in each specialty and year that were classified as generalists by their respective Medicare determined subspecialty label but were reclassified as subspecialists using our CBS labels. Neurosurgery, Ophthalmology and Otolaryngology only have a general subspecialty label across all years.

© 2025 Karadakic R, et al. *JAMA Health Forum*.

Table S6: Summary Statistics of Main Estimation Sample

**Variable**	**Mean**	**SD**	**Min**	**Median**	**Max**
HHI	0.231	0.155	0	0.189	1
Nr. Unique HCPCS	12.65	8.359	1	11	122
Subspecialists per FFS	80.60	20.54	23.03	80.00	221.53
Generalists per FFS	97.82	38.265	14.193	95.261	278.908
Nr. FFS Enrollees	287,906	237,188	17,004	211,672	1,342,105

Notes: This table provides summary statistics of the main variables used for adjusted regression models of the implications of subspecialization on the practice scope of generalists. All five surgical specialty groups (general surgery, neurosurgery, ophthalmology, orthopedic surgery, otolaryngology) are included.

© 2025 Karadakic R, et al. *JAMA Health Forum*.

Table S7: Association of Subspecialists per FFS Beneficiary with Measures of Generalists Scope of Practice (Raw Regression Output)

	Unique Procedures		HHI	
Variable	**(1 – Simple Model)**	**(2 – Restricted Model)**	**(3 - Simple Model)**	**(4 – Restricted Model)**
log(Subspecialists)	−0.149***	−0.094***	0.157***	0.053**
	[−0.165, −0.133]	[−0.148, −0.041]	[0.144, 0.170]	[0.013, 0.092]
log(Generalists)		−0.108+		0.109**
		[−0.221, 0.005]		[0.031, 0.188]
log(FFS Enrollees)		−0.044***		0.029***
		[−0.065, −0.023]		[0.015, 0.043]
Observations	102,013	102,013	101,772	101,772
Mean Dep. Variable	12.65	12.65	0.231	0.231
R2	0.003	0.108	0.006	0.098
Standard Errors	HC1	by: State	HC1	by: State
FE: State		X		X
FE: Year		X		X
FE: Specialty		X		X

Note: The table presents the results of a simple OLS regression of our outcome on the number of subspecialists per 100,000 FFS enrollees (columns 1 and 3). All five surgical specialty groups (general surgery, neurosurgery, ophthalmology, orthopedic surgery, otolaryngology) are included. Results for our main specification are presented in columns 2 and 4. 95% confidence intervals are constructed using heteroskedasticity robust standard errors and where applicable we cluster standard errors at the state level. Significance thresholds+ p < 0.1, * p < 0.05, ** p < 0.01, *** p < 0.001

© 2025 Karadakic R, et al. *JAMA Health Forum*.

eReferences

1. National Uniform Claim Commiettee. Health Care Provider Taxonomy Code Set. Health Care Provider Taxonomy Code Set. 2024. Accessed April 30, 2024. https://taxonomy.nucc.org/
2. Arora A, Dell M. LinkTransformer: A Unified Package for Record Linkage with Transformer Language Models. Published online 2024.
3. Center for Medicare and Medicaid Services. CMS Specialty Code/Healthcare Provider Taxonomy Crosswalk. https://www.cms.gov/Medicare/Provider-Enrollment-and-Certification/MedicareProviderSupEnroll/downloads/taxonomy.pdf.
4. Laurens van der Maaten, Geoffrey Hinton. Visualizing Data using t-SNE. *Journal of Machine Learning Research*. 2008;9:2579-2605.
5. Wattenberg M, Viégas F, Johnson I. How to Use t-SNE Effectively. *Distill*. 2016;1(10). doi:10.23915/distill.00002

© 2025 Karadakic R, et al. *JAMA Health Forum*.
