# Supplementary material for: Subspecialization of Surgical Specialties in the US
Source: JAMA Health Forum. 2025 Sep 19;6(9):e253192. doi: 10.1001/jamahealthforum.2025.3192 (PMC12449727; doi:10.1001/jamahealthforum.2025.3192)
Supplement: Supplement 2. — Data Sharing Statement [file jamahealthforum-e253192-s002.pdf]

Data Sharing Statement

Karadakic. Subspecialization of Surgical Specialties in the US. *JAMA Health Forum*. Published September 19, 2025. doi:10.1001/jamahealthforum.2025.3192

Data
Data available: No
